# Sulfate homeostasis in Atlantic salmon is associated with differential regulation of salmonid‐specific paralogs in gill and kidney

**DOI:** 10.14814/phy2.15059

**Published:** 2021-10-07

**Authors:** Marius Takvam, Elsa Denker, Naouel Gharbi, Harald Kryvi, Tom O. Nilsen

**Affiliations:** ^1^ NORCE Norwegian Research Center NORCE Environment Bergen Norway; ^2^ Department of Biological Science University of Bergen Bergen Norway

## Abstract

Sulfate (${\text{SO}}_{4}^{2‐}$) regulation is challenging for euryhaline species as they deal with large fluctuations of ${\text{SO}}_{4}^{2‐}$ during migratory transitions between freshwater (FW) and seawater (SW), while maintaining a stable plasma ${\text{SO}}_{4}^{2‐}$ concentration. Here, we investigated the regulation and potential role of sulfate transporters in Atlantic salmon during the preparative switch from ${\text{SO}}_{4}^{2‐}$ uptake to secretion. A preparatory increase in kidney and gill sodium/potassium ATPase (Nka) enzyme activity during smolt development indicate preparative osmoregulatory changes. In contrast to gill Nka activity a transient decrease in kidney Nka after direct SW exposure was observed and may be a result of reduced glomerular filtration rates and tubular flow through the kidney. In silico analyses revealed that Atlantic salmon genome comprises a single *slc13a1* gene and additional salmonid‐specific duplications of *slc26a1* and s*lc26a6a*, leading to new paralogs, namely the *slc26a1a* and ‐*b*, and *slc26a6a1* and ‐*a2*. A kidney‐specific increase in *slc26a6a1* and *slc26a1a* during smoltification and SW transfer, suggests an important role of these sulfate transporters in the regulatory shift from absorption to secretion in the kidney. Plasma ${\text{SO}}_{4}^{2‐}$ in FW smolts was 0.70 mM, followed by a transient increase to 1.14 ± 0.33 mM 2 days post‐SW transfer, further decreasing to 0.69 ± 0.041 mM after 1 month in SW. Our findings support the vital role of the kidney in ${\text{SO}}_{4}^{2‐}$ excretion through the upregulated *slc26a6a1*, the most likely secretory transport candidate in fish, which together with the *slc26a1a* transporter likely removes excess ${\text{SO}}_{4}^{2‐}$, and ultimately enable the regulation of normal plasma ${\text{SO}}_{4}^{2‐}$ levels in SW.

## Graphical abstract

This study address Sulfate regulation in Atlantic salmon as they deal with large fluctuations of sulfate during migratory transitions between freshwater and seawater. Our findings support the vital role of the kidney in sulfate excretion through the upregulated slc26a6a1, the most likely secretory transport candidate in fish, which together with the slc26a1a transporter likely removes excess sulfate, and ultimately enable the regulation of normal plasma SO_4_^2–^ levels in SW.

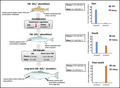

## 1 INTRODUCTION

Fish live in aquatic environments that range from hypo‐osmotic to hyper‐osmotic and are thus more vulnerable to changes in body fluids compared to terrestrial animals (Takei et al., 2014). Teleost fish species regulate salt and water balance through the cooperative efforts of the gills, kidney, and intestine in order to maintain a plasma osmolality range of 300–325 mOsm/kg, irrespective of the environment (Evans et al., 2005; Grosell, 2010; Hickman & Trump, 1969; Marshall & Grosell, 2006; McCormick, Regis, et al., 2013). FW teleosts retain ions by active absorption through the gills and excrete large volumes of water through the kidney to maintain homeostasis in the ion poor environment of FW (3–5 mOsm/kg) (Evans et al., 2005; Marshall & Grosell, 2006; Scott et al., 2005). In contrast, SW teleosts osmotically loose water and passively gain ions from the environment (1000 mOsm/kg) (Evans, 1984; Evans et al., 2005; Marshall & Grosell, 2006). To compensate they need to drink seawater to absorb water in the intestines (Whittamore, 2012), thus loading the blood with NaCl that is actively secreted across gills (Evans, 2010; Hwang et al., 2011; Hiroi & McCormick, 2012; McCormick, Regis, et al., 2013; Takei et al., 2014). In the kidney, minute volumes of iso‐osmotic urine are produced to conserve water (Beyenbach, 2004; Engelund & Madsen, 2015; Hickman & Trump, 1969; Nishimura & Fan, 2003; Nishimura & Imai, 1982) and excess divalent ions (Mg^2+^, ${\text{SO}}_{4}^{2‐}$, Ca^2+^) are secreted (Flik et al., 1996; Chandra et al., 1997; Renfro, 1999; Beyenbach, 2004; Islam et al., 2013, 2014; Kato & Watanabe, 2016). The ion regulatory roles of gills and intestine are well studied in euryhaline fish moving between FW and SW environments (Evans, 2010; Evans et al., 2005; Hiroi & McCormick, 2012; McCormick, Farell, et al., 2013; Grosell, 2010; Sundell & Sundh, 2012; Whittamore, 2012), while knowledge pertaining to the ion regulation mechanisms in the euryhaline teleost kidney is more limited, despite major changes in renal function (transport and filtration rates) are necessary when moving between FW and SW environments (Takvam et al., 2021).

The kidney is especially important for the regulation of ${\text{SO}}_{4}^{2‐}$, as it removes as much as 97% of this ion in SW teleosts (Watanabe & Takei, 2012). Regulation of ${\text{SO}}_{4}^{2‐}$ for euryhaline species is challenging as they deal with large ${\text{SO}}_{4}^{2‐}$ fluctuations when moving between FW (0.3 mM) and SW (30 mM) (Edwards & Marshall, 2012) while maintaining a stable plasma ${\text{SO}}_{4}^{2‐}$ concentration, typically between 0.2 and 1 mM (Watanabe & Takei, 2012). ${\text{SO}}_{4}^{2‐}$ is important for a variety of metabolic and cellular processes, and slight imbalances in plasma ${\text{SO}}_{4}^{2‐}$ levels have been linked to pathological conditions in mammals such as hyposulfatemia, growth retardation, reduced fertility, and seizures (Dawson et al., 2003; Markovich, 2001; Markovich & Aronson, 2007). Hence, high levels of ${\text{SO}}_{4}^{2‐}$ in aquatic environments, particularly in SW, can be toxic for fish if they are unable to efficiently regulate ${\text{SO}}_{4}^{2‐}$ (Elphick et al., 2011). Yet, perturbations in ${\text{SO}}_{4}^{2‐}$ homeostasis may be related to adverse pathological conditions, limited studies have addressed sulfate regulation in teleost (Cliff & Beyenbach, 1992; Kato et al., 2009; Katoh et al., 2006; Pelis & Renfro, 2003; Renfro et al., 1999; Renfro & Pritchard, 1983; Watanabe & Takei, 2011a, 2011b). Based on these investigations ${\text{SO}}_{4}^{2‐}$ are primarily transported (reabsorption or secretion) from proximal tubules in the fish kidney. A complete molecular transport model for ${\text{SO}}_{4}^{2‐}$ has largely been demonstrated in FW‐acclimated Japanese eel (Nakada et al., 2005) and SW‐acclimated Japanese eel (Watanabe & Takei, 2011b), were the solute carrier family 26 (SLC26) and family 13 (SLC13) appears to contribute significantly to ${\text{SO}}_{4}^{2‐}$ regulation in the kidney. However, a complete molecular transport model could not be verified in FW mefugu (Kato et al., 2009). Such species‐specific differences in ${\text{SO}}_{4}^{2‐}$ regulation highlight the requirement for a better understanding across the teleost lineages. Hence, aspects of ${\text{SO}}_{4}^{2‐}$ transport in euryhaline species, especially in FW, still warrants further clarification (Takvam et al., 2021).

Atlantic salmon (*Salmo salar*) is a useful model species due to its anadromous lifecycle migrating between FW and SW environments. Juvenile salmon goes through parr‐smolt transformation (smoltification), during which preparatory osmoregulatory changes transpires in gills, intestine, and kidney, all vital for successful acclimation to seawater (McCartney, 1976; Nilsen et al., 2007, 2008; Tipsmark et al., 2010; McCormick, Regis, et al., 2013; Sundell & Sundh, 2012; Sundell et al., 2014). The salmonid‐specific fourth vertebrate whole genome duplication (Ss4R) results in a large genomic reorganization, highlighting the relevance and significance of Atlantic salmon from an evolutionary perspective (Lien et al., 2016). Salmonids often have paralog genes that adopt a similar or new function in relation to the ancestral gene (Houston & Macqueen, 2019), and paralog retention rate can range between 25% and 75% (Bailey et al., 1978). Genome duplication events can generate new genetic material for mutation, drift, and selection to act upon, promoting phenotypic diversity (Kellogg, 2003; Kondrashov et al., 2002), which suggest an important role for paralog genes (duplicates) in explaining the remarkable plasticity of salmon adapting to different environments. The purpose of this study was to 1) identify key ${\text{SO}}_{4}^{2‐}$ transporters in the salmon genome and their tissue distribution and 2) determine changes in ${\text{SO}}_{4}^{2‐}$ transporters expression during smoltification and SW acclimation.

## 2 MATERIALS AND METHODS

### 2.1 Fish material, experimental design, and sampling

On September 4, juvenile Atlantic salmon (*Salmo salar* L.) parr (average weight 30 grams) of AquaGen stock were obtained from the Aquatic Laboratory of Bergen (ILAB) and haphazard distributed into the experimental tanks. The fish was reared under conditions similar to standard commercial production protocols and are therefore exempt from the Norwegian Regulation on Animal Experimentation (NARA). The control group (parr) was kept under 12‐h darkness and 12‐h light (12D:12L; winter signal) photoperiod regime during the whole experimental period while the other experimental group (smolt) was given a 24‐h light regime (24L) resulting in a classic square wave photoperiodic induction of smoltification (Stefansson et al., 1991). Both groups had similar tank environment (1m^3^, 400 l rearing volume) and kept in freshwater (*Salinity*; 1%–2‰, *Temp*; 10 ± 0.23℃, *oxygen outlet water*; >80%, and *Flow rate*; 0.6 l/kg/min). Fish were fed by automatic feeders to satiation during the 12‐h light phase. FW smolts were transferred to SW (1 m^3^ 160 l rearing volume: *Salinity*; 32 ‰, *Temp*; 9.2 ± 0.3℃, *Oxygen outlet water*; <80%, and *Flow rate*; 0.6 l/kg/min) on the October 20 while the parr (control) was kept in FW. Sulfate concentrations in the experimental FW and SW were 0.1 mM and 33 mM, respectively.

After the 24L regime was initiated, tissue samples were collected after 12 days (120 day degrees; d.d (number of days × mean temperature)), 26 days (260 d.d), 35 days (350 d.d), and 45 days (450 d.d) in FW. Parr (control) kept in FW were also sampled after 83 days (830 d.d). Smolts transferred to SW were sampled at 1 day (480 d.d), 2 days (490 d.d), and 38 days after SW transfer (830 d.d). For each sampling, juveniles (12 individuals per group) were quickly dip‐netted out of the tanks and anesthetized using a lethal dose of tricaine methanesulfonate (100 mg l^−1^ MS222; Sigma, St Louis, MO, USA). Blood was collected from the caudal vein and stored on ice until centrifugation (4℃, 3000 g, 5 min) and plasma aliquots were frozen. Fork length and body weight were measured, before gills, kidney, and intestine were dissected out and preserved in different media depending on later applications. Condition factor was calculated according to the Fulton's formula *CF* = *L^3^* (Nash et al., 2006) and each fish was given a smolt score/index ranging from 1 to 5 based on the criteria outlined in Table S1 (see Figure S1). At all representative timepoints, samples were preserved and stored as follows (1) for Nka activity measurement: SEI buffer (250 mM sucrose, 10 mM Na_2_EDTA, and 50 mM imidiazole) −80℃ (gills/kidney) and (2) for mRNA expression analysis: first overnight at 4℃ in RNAlater, then transferred to −80℃ (gills/kidney/intestine).

### 2.2 Plasma sulfate concentrations and Nka enzyme activity (gills, kidney)

Plasma sulfate concentrations were determined using the sulfate assay kit (Quantichrom^TM^ Sulfate Assay Kit, DSFT‐200) according to the protocol described by the manufacturer (Bioassay system, 3191 Corporate Place, Hayward, CA 94545, USA). The method utilizes the quantitative formation of insoluble barium sulfate (BaSO_4_) in polyethylene glycol and the absorbance was measured on a Spark multimode microplate reader (Tecan, Mannedorf, Switzerland) at 600 nm (room temperature, endpoint measurement). The same protocol was used to determine sulfate concentrations in FW and SW.

Nka enzyme activity was assessed according to the microassay method of McCormick (1993). Briefly, Nka activity was measured in gill filaments (*n* = 4–6) and kidney tissue (0.5–1 mg, see Supplementary data Figures S2 and S3). The reaction is enzymatically coupled with the oxidation of nicotinamide adenine dinucleotide (NADH) by pyruvate kinase and lactic dehydrogenase, which could be directly measured on a Spark multimode microplate reader at 340 nm (25ºC, 60 cycles, 10 min). The protein concentration was determined using the Pierce BCA Protein Assay kit (Thermo Fisher Scientific, Massachusetts, USA) measuring the absorbance at 562 nm in the Spark multimode microplate reader. The final Nka enzyme activity is reported as µmoles ADP per mg protein per hour.

### 2.3 Identification of sulfate (${\text{SO}}_{4}^{2‐}$) transporters in the Atlantic salmon genome

Atlantic salmon (*Salmo salar*) Slc13a1, Slc26a1, and Slc26a6 sequences were identified by a BLAST search in the National Center for Biotechnology information (NCBI) database using known Japanese eel (*Anguilla japonica)* and medaka (*Takifugu obscurus*) protein sequences. For each of the transporter families, Atlantic salmon sequences were aligned using the CLUSTALW algorithm in Seaview (http://doua.prabi.fr/software/seaview) with already annotated genes from teleost species, representing the diversity of the group (including the Japanese eel and the medaka), as well as representative species from all vertebrate groups. The most informative residues of the alignment were selected by the Gblocks tool (included in Seaview), using default parameters. The resulting new alignment was then submitted to a maximum‐likelihood phylogenetic analysis using PhyML (also in Seaview) (nearest neighbor interchanges; NNI) and node support was calculated using a Bootstrap analysis (100 replicates). The resulting phylogenetic trees were formatted using the FigTree tool software. To confirm the identity of the gene candidates, especially in cases when salmon‐specific duplications were suspected, a synteny analysis was applied. The principle was to verify the salmon‐specific duplication by comparing it with the Northern pike (*Esox lucius*), a closely related species to Atlantic salmon that did not undergo a fourth round of whole genome duplication (Ss4R). The gene environment for each transporter in the pike was visualized using Genomicus (https://www.genomicus.biologie.ens.fr/genomicus‐99.01/cgi‐bin/search.pl) and the genes around the salmon transporters were manually analyzed on the genome browser of NCBI. The figure was then made using Inkscape (Figure 4).

### 2.4 RNA isolation and cDNA synthesis

Approximately 20–25 mg of kidney and gill tissue were homogenized in 600 µl of RLT plus buffer and Reagent DX (Qiagen QIAsymphony mRNA extraction kit) using ceramic spheres and the Precellys 24 tissue homogenizer (Bertin Technologies, Montigny‐le‐Bretonneux, France). Total RNA was extracted using the QIAsymphony Robot (Qiagen) and the QIAsymphony RNA kit, following the manufacturer's protocol (Qiagen). Isolated total RNA was eluted in 100 µl (kidney) and 50 µl (gills) of ultra‐pure water and stored at −80℃. Quantification of RNA concentrations for kidney and gill tissue was performed using the Invitrogen Qubit 4 Fluorometer (Thermo Fisher Scientific) applying the Qubit^TM^ RNA HS Assay Kit protocol (Invitrogen^TM^, Thermo Fisher Scientific). Sufficient integrity of total RNA was validated using Agilent RNA 6000 Nano kit and Agilent 2100 expert analyzer (Agilent technologies). cDNA was synthesized using 1500 ng (kidney) and 500 ng (gills) total RNA and Oligo(dT)_20_ primer in conjunction with SuperScript^™^ III Reverse Transcriptase kit (Invitrogen, Oslo) according to the manufacturer's instructions.

### 2.5 Tissue distribution and temporal gene expression profile using real‐Time qPCR

Real‐time quantitative PCR (qPCR) was carried out using iTaq^™^ Universal SYBR^®^ Green Supermix (Bio‐Rad Laboratories) in a total volume of 12.5 µl, using exon junction‐spanning primers (Table 1) at final concentration of 200 nM. The reactions were run in a C1000 Touch^™^ Thermo cycler, CFX96^™^ Real‐Time PCR detection System, and CFX Manager software (software version 3.1; Bio‐Rad Laboratories). The thermal conditions consisted of an initial denaturation for 2 min at 95℃, followed by 37 cycles at 95℃ for 15 s and 60℃ for 25 s. Melt curve analysis verified that the primer sets for each qPCR assay had no primer–dimer artifacts and generated only one single product.

**TABLE 1:** Target and reference genes/primers for tissue distribution and qPCR

Gene	Primer forward (5′−3′)	Primer reverse (5′−3′)	mRNA reference
slc26a1a	GTAGAGCGAGTTGGTTGTGAGG	GCTGTGCTCCCACACTTCG	XM_014129156.1
slc26a1bX1	GTTGGCTGTAAGTGTGAGGGAC	CCTCTGGAAGTGGTAGGCTG	XM_014138168.1
slc26a1bX3	GTGACACATGTTGGCTGAGCAC	GCTTCGTCTTCAGGATGGCC	XM_014138170.1
slc26a6a1	CTCATCTCCTACTACGGCAACCTG	CTGGGAGACTTCAGCCCTCTG	XM_014134693.1
slc26a6a2	GACCTGAAATTGAACCAGACGGCC	GTGTGTGTCGTTGACGGAGTTC	XM_014192131.1
slc26a6b	ACAGAGAGGTGCTGGATGAGGG	GGGGACAGAACACCTCACTGAC	XM_014135170.1
slc26a6c	GTACTGGATGAGCAGAGACTGGAGG	GCCTGGGTACAGTACATCTGAAGGACTC	XM_014132723.1
slc13a1	ACCCTCTCAGACCAATGCGATTGG	GGAAGGGTGGCAATCCCTCCTATAGAG	XM_014169986.1
ef1a	CCCTGTGGAAGTGGCTGAAG	CATCCAAGGGTCCGTATCTCTT	Olsvik et al. (2013)

Overview of primer sequences used for tissue distribution and to measure mRNA abundance of target genes slc26a1a, slc26a1bX1, slc26a1bX3, slc26a6a1, slc26a6a2, slc26a6b, slc26a6c, slc13a1, and the reference gene ef1a.

Tissue distribution analysis was performed in gills, kidney, urine bladder, liver, and intestine from FW‐acclimated salmon (*n* = 3) and SW‐acclimated salmon (*n* = 3). Genes not detected or expressed at Ct values >30 (Bustin et al., 2009) were not used for further analysis (see Supplementary Data; Table S2). Hence, the s*lc26a1a*, s*lc26a1b* isoforms *X1* and *X3*, *slc26a6a1*, *slc26a6b*, and *slc26a6c* in all kidney samples and the *slc26a6a2* in all gill samples were quantified during smoltification and after SW transfer (Table 1) using cDNA dilutions of 1:20 (gills, 25 ng/µl) and 1:30 (kidney, 50 ng/µl). Validation of the endogenous reference gene(s) *gapdh*, *ef1a*, and *b*‐*actin* was conducted using the RefFinder (Xie et al., 2012): BestKeeper (Pfaffl et al., 2004), NormFinder (Andersen et al., 2004), Genorm (Vandesompele et al., 2002), and the comparative delta‐Ct method (Silver et al., 2006). The *ef1a* was determined as the most stable reference gene for normalization in both kidneys and gills. Relative expression was calculated according to the PCR efficiency corrected formulas from Pfaffl (2001).

### 2.6 Statistical analysis

All statistical analysis were performed using RStudio (RStudio version 1.2) utilizing the following packages: Rtools, dplyr, ggplot2, car, and emmeans. Statistical differences were determined either by linear models (two‐way ANOVA) or a generalized linear model (glm) for non‐normal response (family: Gamma and Gaussian) followed by a Tukey's HSD post hoc test. *p* values lower than 0.05 (*p* < 0.05) were deemed a statistically significant datapoint and marked with asterisk (between groups) accordingly; *p* < 0.05 (*), *p* < 0.01 (**), and *p* < 0.001 (***). Non‐identical letters were used for significant difference between timepoints/samplings in each group. Results are presented as mean ± the standard error of mean (SEM).

## 3 RESULTS

### 3.1 Osmoregulatory activity in gills and kidney

**FIGURE 1:**
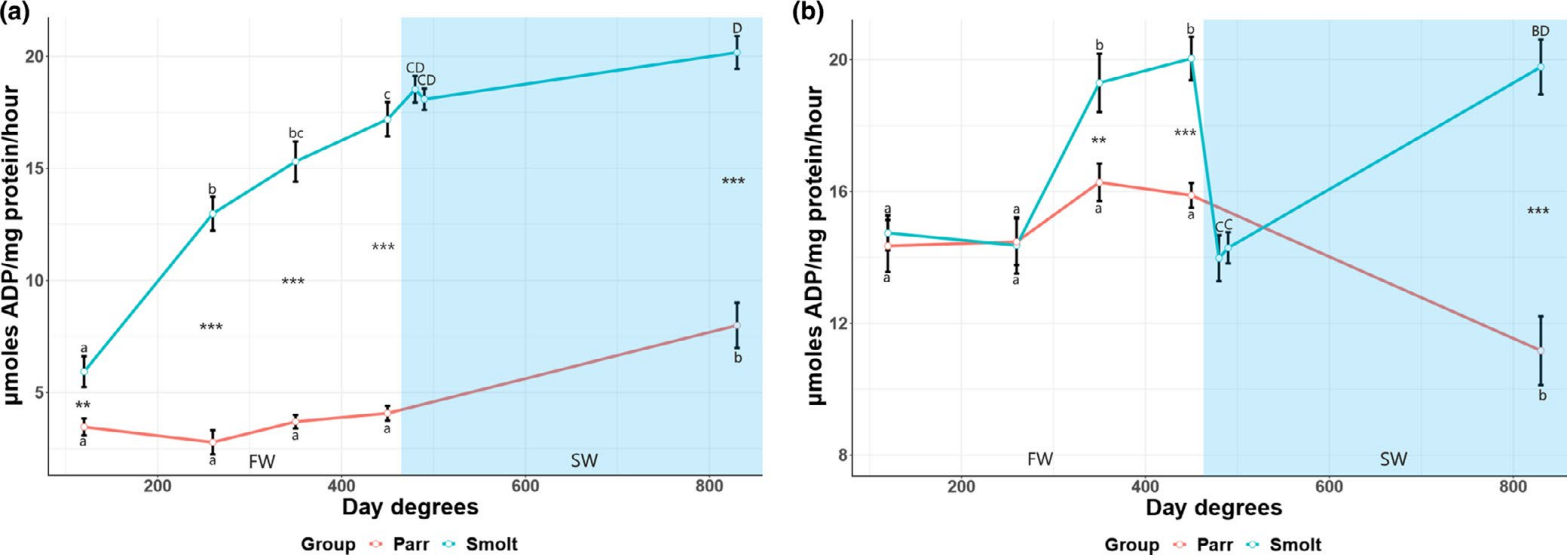
Gill (a) and Kidney (b) Nka enzyme activity levels (µmoles ADP/mg protein/hour) of juvenile Atlantic salmon parr and smolts in freshwater (FW) and smolts after seawater (SW) transfer. Different small letters indicate significant differences between timepoints within the control group (parr) and experimental smolt group in FW (white area of graph), while capital letters indicate differences within each group in SW (blue area of graph). Note that significances following SW transfer are related to last timepoint in FW. Asterisk *p < 0.05, **p < 0.01, and ***p < 0.001 indicate significant differences between groups at each timepoint in both FW and SW. The control group remained in FW during the entire experiment. Each data point is represented as mean ± Standard Error of Mean (SEM) and *n* = 10–12

Gill Nka enzyme activity levels in the smolt group increased from 5.93 ± 0.68 µmoles ADP/mg protein/h after 12 days (120 day degrees (d.d)) to 12.98 ± 0.75 after 26 days (260 d.d), reaching peak activity levels of 17.19 ± 0.76 µmoles ADP/mg protein/hour after 45 days (450 d.d) in FW (Figure 1a). Gill Nka activity was slightly elevated after 38 days in SW (20.17 ± 0.73). In the parr group, gill Nka enzyme activity levels remained low until a significant increase to 7.99 ± 1 µmoles ADP/mg protein/hour after 83 days (830 d.d) during the FW phase (Figure 1a). The smolt group displayed consistently higher gill enzyme activity levels than those observed in the parr group (Figure 1a).

Initial kidney Nka enzyme activity levels in the smolt group (14.47 ± 0.70 µmoles ADP/mg protein/hour) increased to 19.30 ± 0.88 after 35 days (350 d.d), reaching peak levels of 20.03 ± 0.66 µmoles ADP after 45 days (450 d.d) in FW (Figure 1b). Kidney Nka activity in smolts rapidly decreased to 13.98 ± 0.70 after 1 day in SW and remaining low (14.29 ± 0.47 µmoles ADP) after 2 days in SW followed by a significant increase to 19.78 ± 0.83 µmoles ADP (Figure 1b). In the parr group, kidney Nka enzyme activity levels remained stable around approximately 14–15 µmoles ADP until a significant decrease to 11.17 ± 1.04 µmoles ADP after 83 days (830 d.d) in FW (Figure 1b), resulting in a significant lower kidney Nka enzyme activity level than those observed in the smolt group after 35 days in FW (350 d.d), 45 days in FW (450 d.d), and 38 days in SW (830 d.d) (Figure 1b).

### 3.2 Plasma sulfate (${\text{SO}}_{4}^{2‐}$) concentration

**FIGURE 2:**
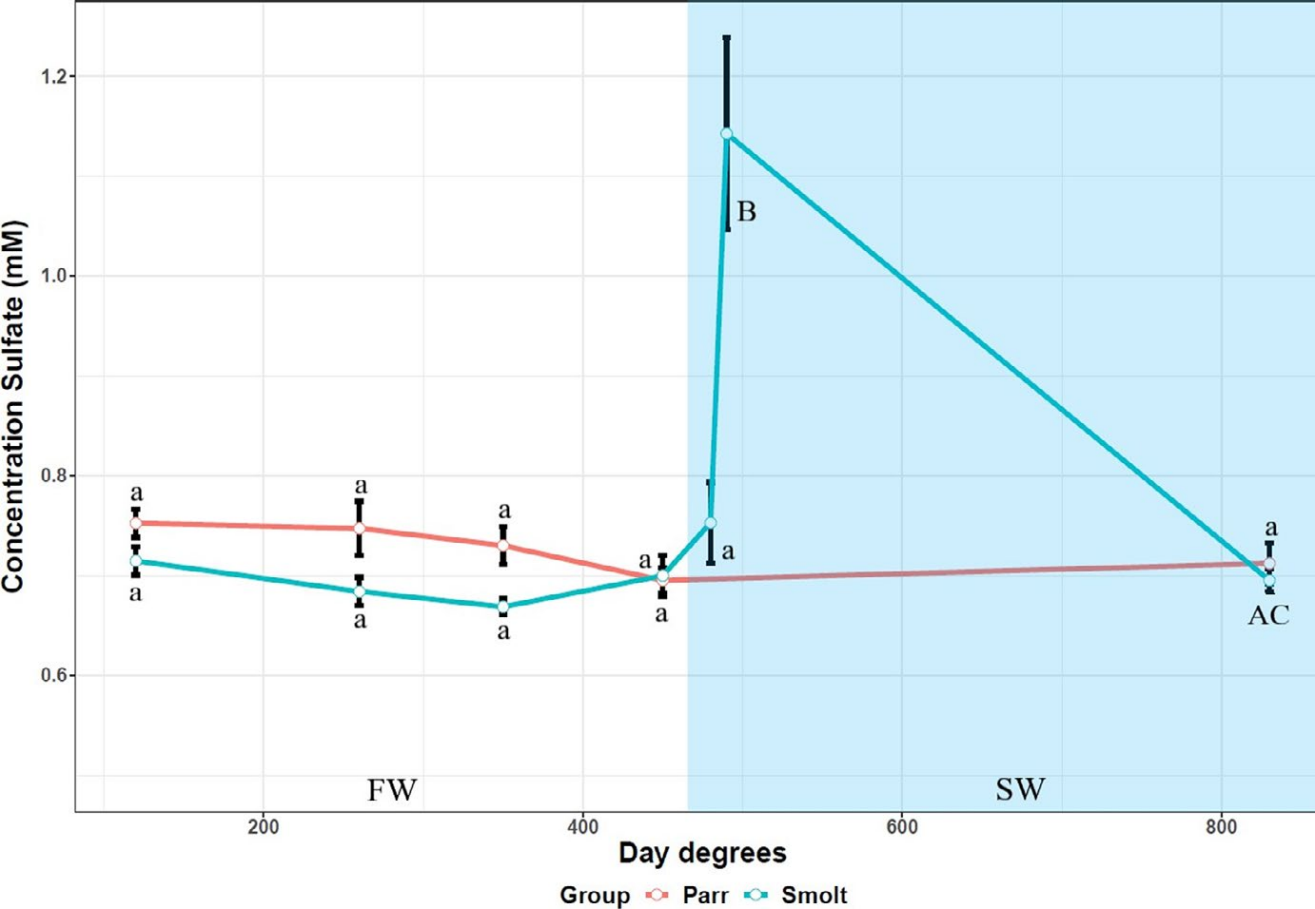
Plasma sulfate levels of juvenile Atlantic salmon parr and smolts in freshwater (FW) and smolts after seawater (SW) transfer. Different small letters indicate significant differences between timepoints within the control group (parr) and experimental smolt group in FW (white area of graph), while capital letters indicate differences within each group in SW (blue area of graph). Note that significances following SW transfer are related to last timepoint in FW. Asterisk *p < 0.05, **p < 0.01, and ***p < 0.001 indicate significant differences between groups at each timepoint in both FW and SW. The control group remained in FW during the entire experiment. Each data point is represented as mean ± Standard Error of Mean (SEM) and *n* = 10–12

Plasma ${\text{SO}}_{4}^{2‐}$ levels remained stable in both the parr (0.72 ± 0.03 millimolar; mM) and smolt (0.70 ± 0.04 mM) during the FW phase (Figure 2), while plasma ${\text{SO}}_{4}^{2‐}$ in the smolt group increased to 1.14 ± 0.33 mM after 2 days in SW (480 d.d), returning back down to 0.69 ± 0.02 mM after 1 month in SW, similar to levels at last timepoint in FW (450 d.d, 45 days, FW) and the parr group (control, 830 d.d, FW) (Figure 2). No significant difference in plasma ${\text{SO}}_{4}^{2‐}$ levels was observed between parr and smolt in FW.

### 3.3 The Atlantic salmon ${\text{SO}}_{4}^{2‐}$ transporter repertoire

**FIGURE 3:**
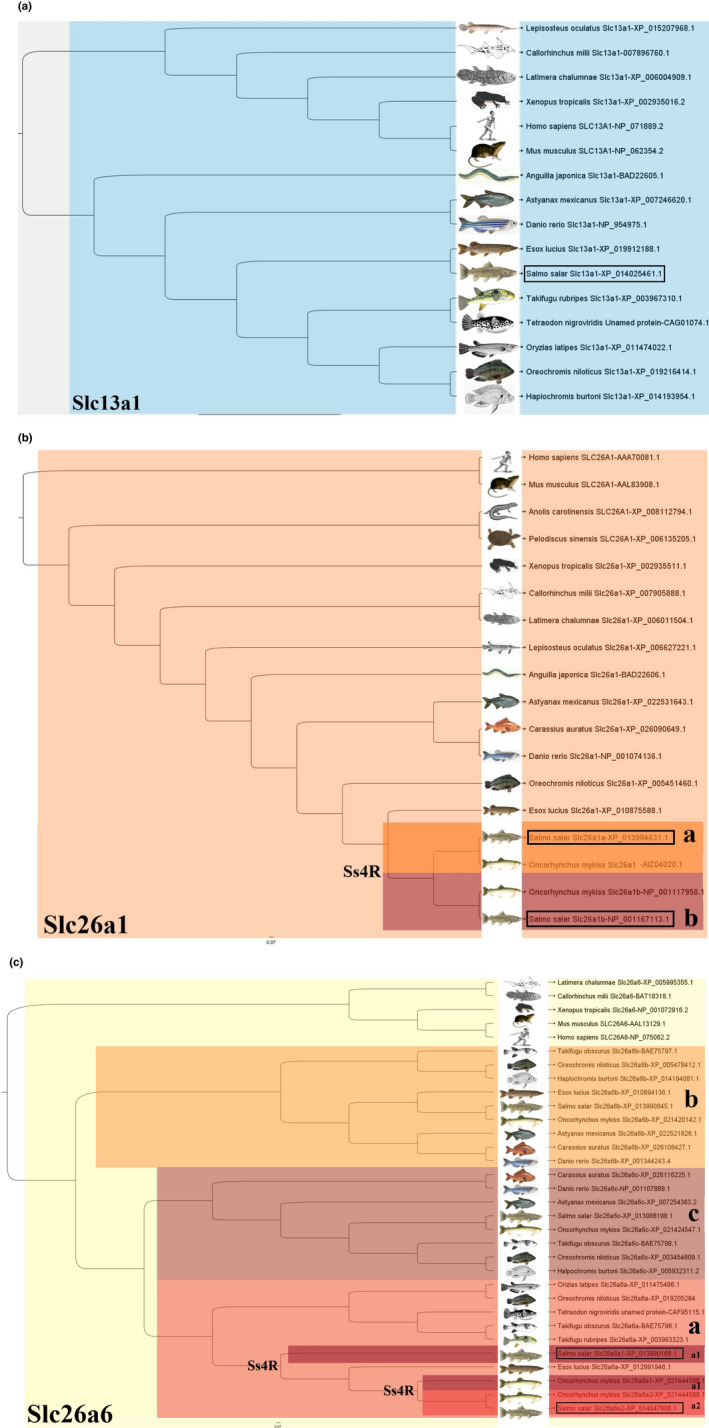
Phylogenetic analysis placing the Atlantic salmon candidate for a Slc13a1 (a), Slc26a1 (b), and Slc26a6 (c) homolog within the Slc13a1, Slc26a1, and Slc26a6 family. Phylogenetic tree presenting the phylogenetic relationship between the protein sequences of Slc13a1, Slc26a1, and Slc26a6 of Atlantic salmon (Salmo salar) and a set of other vertebrate species, using Figtree as graphical viewer. In the Slc13a1 only a single salmon sequence grouped within the vertebrate Slc13a1 group was detected, while in the Slc26a1 family two sequences appeared to be paralogs and were named Slc26a1a (dark orange) and Slc26a1b (red). In the Slc26a6 family three sequences were found and were named Slc26a6b, Slc26a6c, and Slc26a6a. In the latter, two salmon Slc26a6a paralogs aligned with rainbow trout and were named Slc26a6a1 and Slc26a6a2. The salmon‐specific fourth round of whole genome duplication is marked (Ss4R). Protein sequences from fish species are written with first letter upper case (Slc13a1, Slc26a1, and Slc26a6), whereas mammals are written with all letters in upper case (SLC13A1, SLC26A1, and SLC26A6). Pictures of all species are retrieved from public domain or fish base (www.fishbase.org)

The phylogenetic analysis of salmon solute carrier family 13 member 1 (Slc13a1), solute carrier family 26 member 1 (Slc26a1), and member 6 (Slc26a6) orthologues is presented in Figure 3a–c (the corresponding protein alignment is presented in the Supplementary Data; Figures S4–S6). A single salmon sequence grouped within the vertebrate Slc13a1 group, with the closest relative being the Northern pike (*Esox lucius*) Slc13a1––a position consistent with the evolutionary relationship between these species. The putative salmon Slc26a1a and Slc26a1b sequences grouped within the vertebrate Slc26a1, each pairing with the rainbow trout (*Oncorhynchus mykiss)* sequences Slc26a1a and Slc26a1b, with the group containing these four sequences being the closest relative to the single Northern pike (*Esox lucius*) Slc26a1. This position was consistent with an evolutionary relationship between the Northern pike and indicated that the two salmon sequences, named Slc26a1a and Slc26a1b, are the result of a salmonid‐specific duplication. Of the four salmon sequences grouped within the vertebrate Slc26a6, one cluster in a subgroup containing *Takifugu obscurus* Slc26a6b, one aligns in a subgroup containing *Takifugu obscurus* Slc26a6c, and two aligned in a subgroup containing *Takifugu obscurus* Slc26a6a. In the latter, two salmon Slc26a6a paralogs aligned with rainbow trout were named Slc26a6a1 and Slc26a6a2 and these four sequences had as closest relative to the single Northern pike Slc26a6a.

**FIGURE 4:**
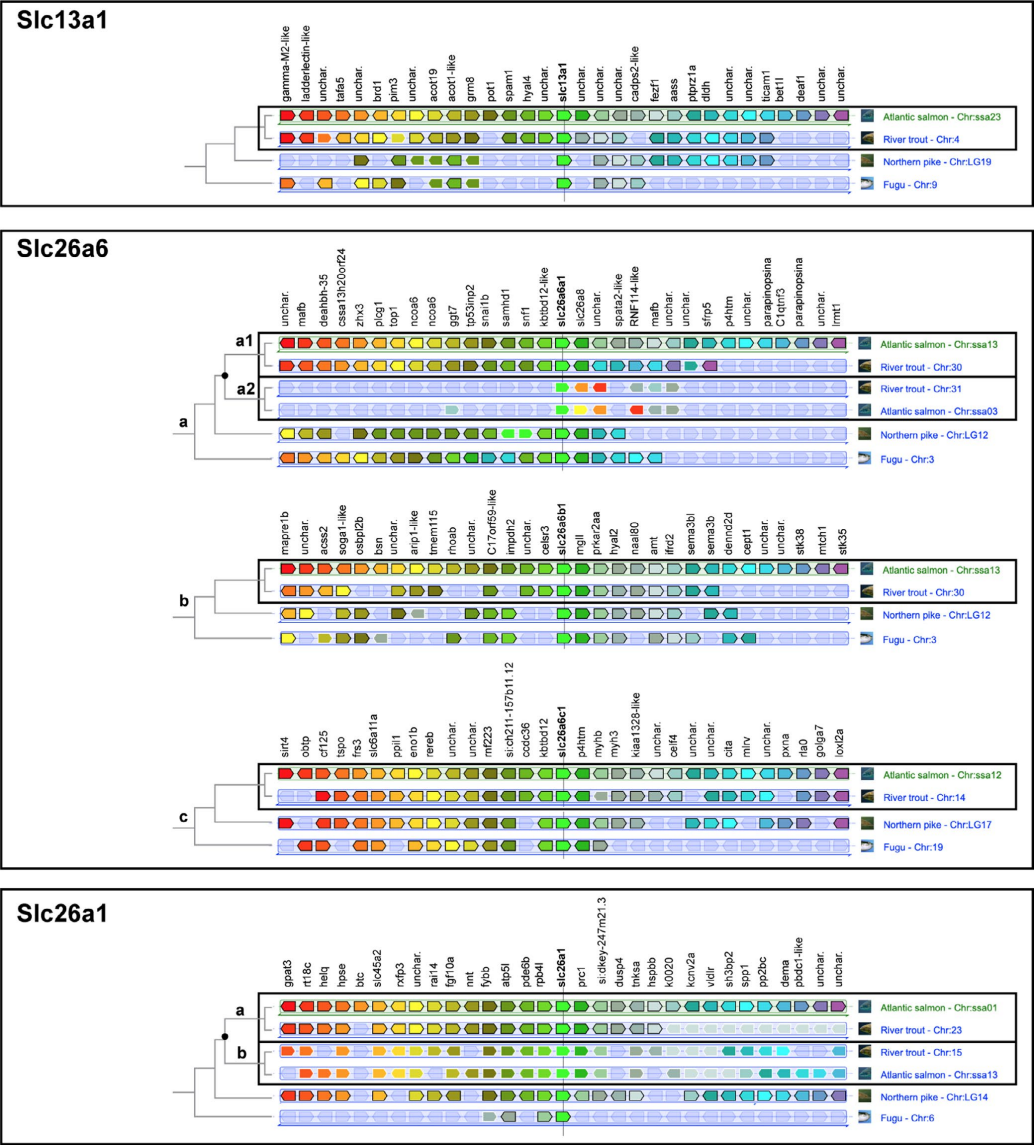
Gene identity confirmation for Slc13a1, Slc26a1, and Slc26a6 protein family. A synteny analysis was performed, comparing the chromosomal arrangement of genes surrounding the Slc13a1, Slc26a1, and Slc26a6 genes. The gene environment for each transporter was retrieved using Ensembl genome browser annotations via the Genomicus platform (Nguyen et al., 2018), complemented with salmon sequences from the NCBI GenBank when not available in Ensemble. Genomicus and the genes around the salmon transporters were manually analyzed on the genome browser NCBI and subsequently visualized with Inkscape. The synteny results concluded that Atlantic salmon underwent a fourth round of genome duplication not observed in the closely related Northern pike (Esox lucius). Paralogs were detected for slc26a1 (slc26a1a and slc26a1b) and slc26a6a (slc26a6a1 and slc26a6a2) in the Atlantic salmon genome. The genes surrounding the candidate genes (Slc13a1, Slc26a6, and Slc26a1) have been given different colors to better differentiate between the genes described. The black spot in the slc26a6a and slc26a1 family indicate the fourth round of genome duplications and the strikethrough lines through the colored boxes highlight the candidate genes

To confirm the orthology and paralogy assignment inferred by the phylogenetic analysis, the chromosomal environment of the Atlantic salmon Slc13a1, Slc26a1, and Slc26a6 paralogs was analyzed to establish syntenic relationships (Figure 4). For *slc13a1*, *slc26a6b*, and ‐*c*, which did not show additional salmonid‐specific paralogs neither in Atlantic salmon nor river trout, a clear homology between the gene environment in the single salmon, trout, Northern pike, and mefugu, further support the phylogenetic relationship between them. For the *slc26a6a* and *slc26a1a*, for which we identified two salmonid paralogues (*slc26a6a1* and −*2*, and *slc26a1a* and ‐*b*), a clear homology between the gene environment of both salmonid paralogs and their single Northern pike and mefugu counterparts, further supports that both salmonid duplicates are orthologues of other teleosts genes, and are paralogs resulting from the fourth round of genome duplication underwent by salmonids.

### 3.4 Tissue distribution of ${\text{SO}}_{4}^{2‐}$ transporters in gills, intestine, kidney, liver, and urinary bladder

Tissue distribution of the Atlantic salmon homologs *slc13a1*, *slc26a1a*, *slc26a1bX1*, *slc26a1bX3*, *slc26a6a1*, *slc26a6a2*, *slc26a6b*, *and slc26a6c* transporters was examined for both FW‐ and from SW‐acclimated salmon.

**FIGURE 5:**
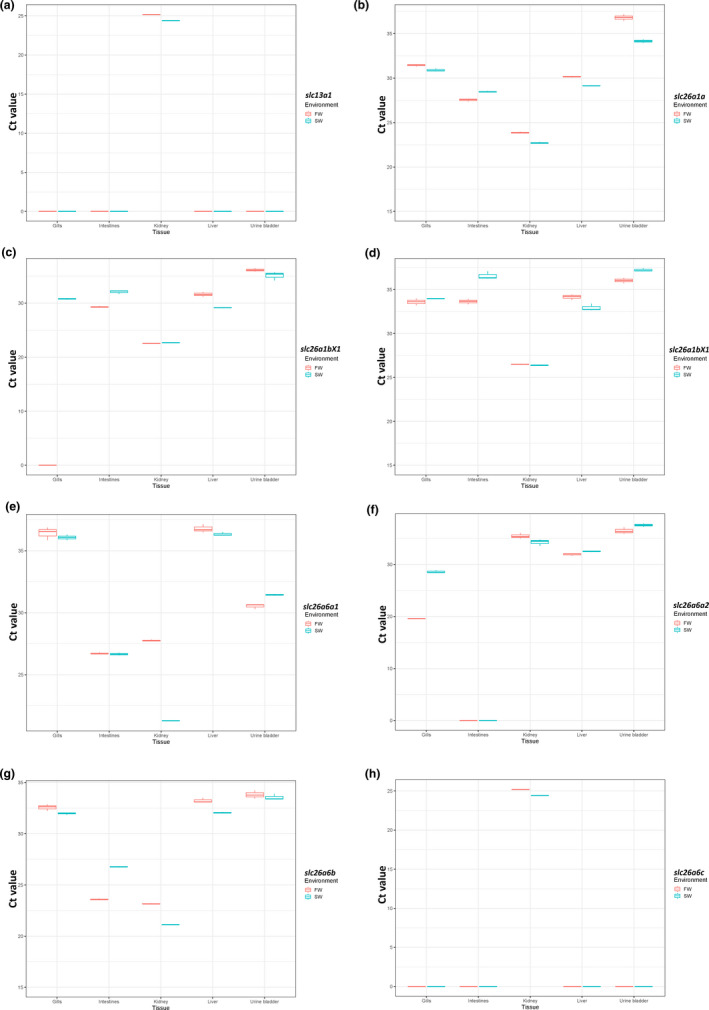
Tissue distribution of the slc13a1 (a), slc26a1a (b), slc26a1bX1 (c), slc26a1bX3 (d), slc26a6a1 (e), slc26a6a2 (f), slc26a6b (g), and slc26a6c (h). Threshold cycles (Ct) in intestine, kidney, liver, and urinary bladder indicate tissue‐specific distribution and mRNA abundance putative sulfate transporters distribution in FW‐acclimated Atlantic salmon (red) and SW‐acclimated Atlantic salmon (blue). Each individual tissue is based on Ct values from triplicate reactions from cDNA pool of three individual salmon (*N* = 3) in FW and SW

The *slc13a1* was only detected in the intestine (Figure 5a), while the *slc26a1a* and *slc26a1bX1* were detected at physiological relevant levels (Ct < 30) in both kidney and intestine (Figure 5b and c). The *slc26a1bX3* was only expressed in the kidney (Figure 5d). No clear regulation between FW and SW was observed for these four genes.

The *slc26a6a1* was detected in intestine and kidney only in SW‐acclimated salmon (Figure 5e). By contrast, *slc26a6a2* paralog was only detected in gills, being particular abundant in FW salmon (Figure 5f). The *slc26a6b* presented a pattern similar to *slc26a1a* and *slc26a1bX1*, while the *slc26a6c* and *slc26a1bX3* only was detected in the kidney, with no clear difference between FW‐ and SW‐acclimated salmon (Figure 5f, g, and h). Based on the above expression patterns, we decided to pursue genes expressed in kidney and gill during smoltification.

### 3.5 ${\text{SO}}_{4}^{2‐}$ transporter mRNA abundance in kidney during smoltification and sea water transfer

**FIGURE 6:**
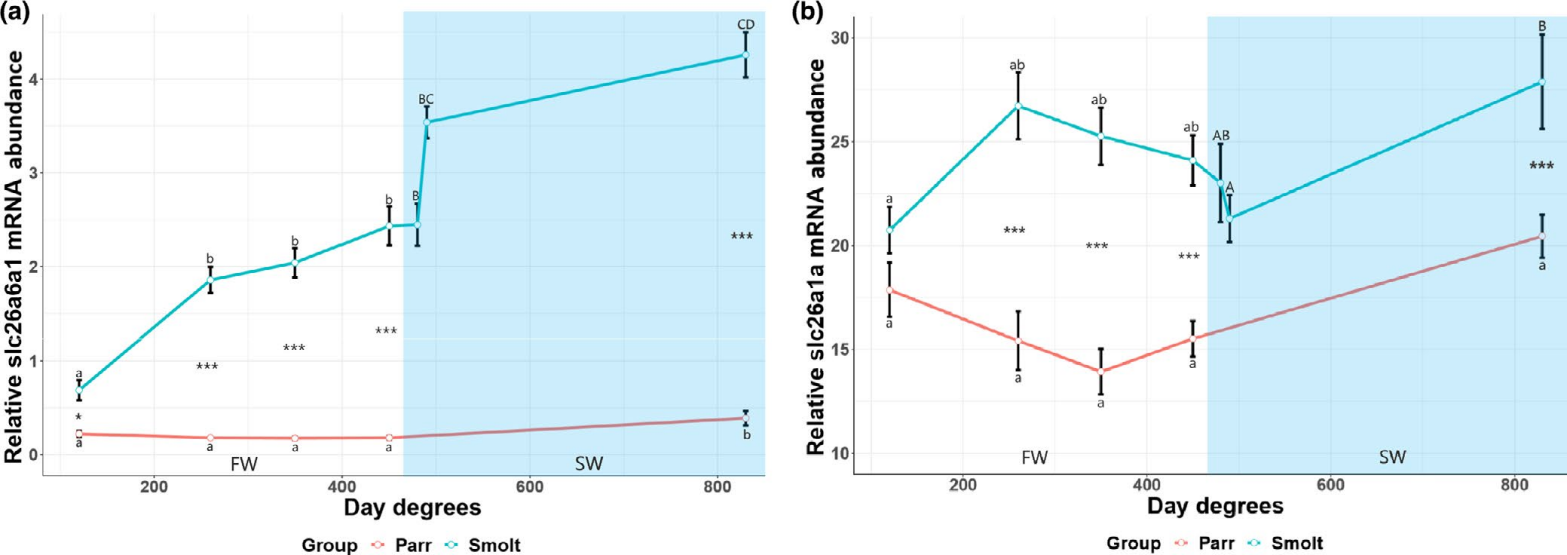
The mRNA abundance of slc26a6a1 (a) and slc26a1a (b) in the kidney of juvenile Atlantic salmon parr and smolts in freshwater (FW) and smolts after seawater (SW) transfer. Different small letters indicate significant differences between timepoints within the control group (parr) and experimental smolt group in FW (white area of graph), while capital letters indicate differences within each group in SW (blue area of graph). Note that significances following SW transfer are related to last timepoint in FW. Asterisk *p < 0.05, **p < 0.01, and ***p < 0.001 indicate significant differences between groups at each timepoint in both FW and SW. The control group remained in FW during the entire experiment. Each data point is represented as mean ± Standard Error of Mean (SEM) and *n* = 10

In the smolt group, *slc26a6a1* mRNA abundance increased significantly during smoltification, with expression levels being fourfold higher after 45 days (450 d.d) (Figure 6a). After SW transfer, smolts displayed a rapid 0.6‐fold increase after 2 days, with expression levels being twofold higher after 38 days in SW compared to last timepoint in FW (Figure 6a). In contrast, in the parr group, relative *slc26a6a1* mRNA abundance remained low through the whole experiment, until a slight, albeit significant, twofold increase between day 45 (450 d.d) and 83 (830 d.d) (Figure 6a). After 38 days in SW (830 d.d) the smolt group displayed consistently higher *slc26a6a1* mRNA abundance, with an 18‐fold higher expression in smolts than in parr after 83 days in FW (830 d.d).

The *slc26a1a* mRNA levels were highly expressed in both smolt and parr, with *slc26a1a* abundance being consistently higher than those observed in the parr, except on day 12 (120 d.d) (Figure 6b). The elevated *slc26a1a* expression during smoltification remained high after SW transfer, reaching peak expression levels after 38 days in SW (Figure 6b). In contrast, the relative *slc26a1a* mRNA abundance was not significantly different at any timepoint in the parr group (Figure 6b).

**FIGURE 7:**
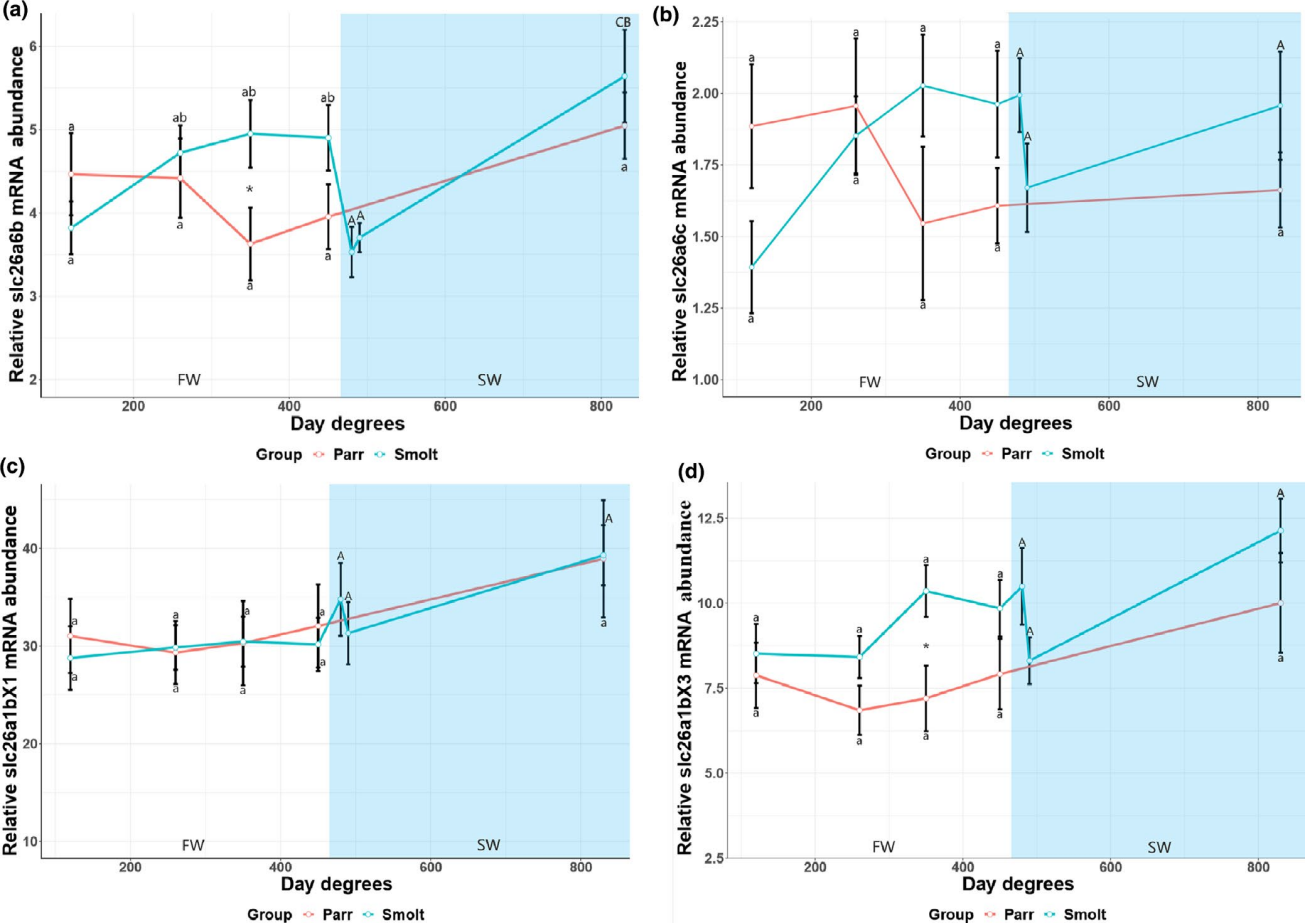
The relative mRNA abundance of slc26a6b (a) and slc26a6c (b), slc26a1bX1 (c) and slc26a1bX3 (d) in the kidney of juvenile Atlantic salmon parr and smolts in freshwater (FW) and smolts after seawater (SW) transfer. Different small letters indicate significant differences between timepoints within the control group (parr) and experimental smolt group in FW (white area of graph), while capital letters indicate differences within each group in SW (blue area of graph). Note that significances following SW transfer are related to last timepoint in FW. Asterisk *p < 0.05, **p < 0.01, and ***p < 0.001 indicate significant differences between groups at each timepoint in both FW and SW. The control group remained in FW during the entire experiment. Each data point is represented as mean ± Standard Error of Mean (SEM) and *n* = 10

The *slc26a6b* and *slc26a6c* were relatively equally expressed in the kidney for both the smolt and parr groups, hence no significant difference was observed in either the smolt group or the parr group (Figure 7a and b). A small but significant difference was observed between groups in relative mRNA abundance of *slc26a6b* after 35 days (350 d.d) in FW (*p *< 0.0214) (Figure 7a and b).

Expression levels of the *slc26a1bX1* (31.04–39.31) and *slc26a1bX3* (7.19–12.13) splice variants were high in the kidney for both the smolt and parr groups, with no significant difference observed in either smolt group or the parr group (Figure 7c and d). A small, yet significant higher *slc26a1bX3* mRNA abundance was observed in the FW smolt group than in the corresponding parr group at day 35 (350 d.d) (Figure 7c and d).

### 3.6 Slc26a6a2 mRNA abundance in gills during smoltification and sea water transfer

**FIGURE 8:**
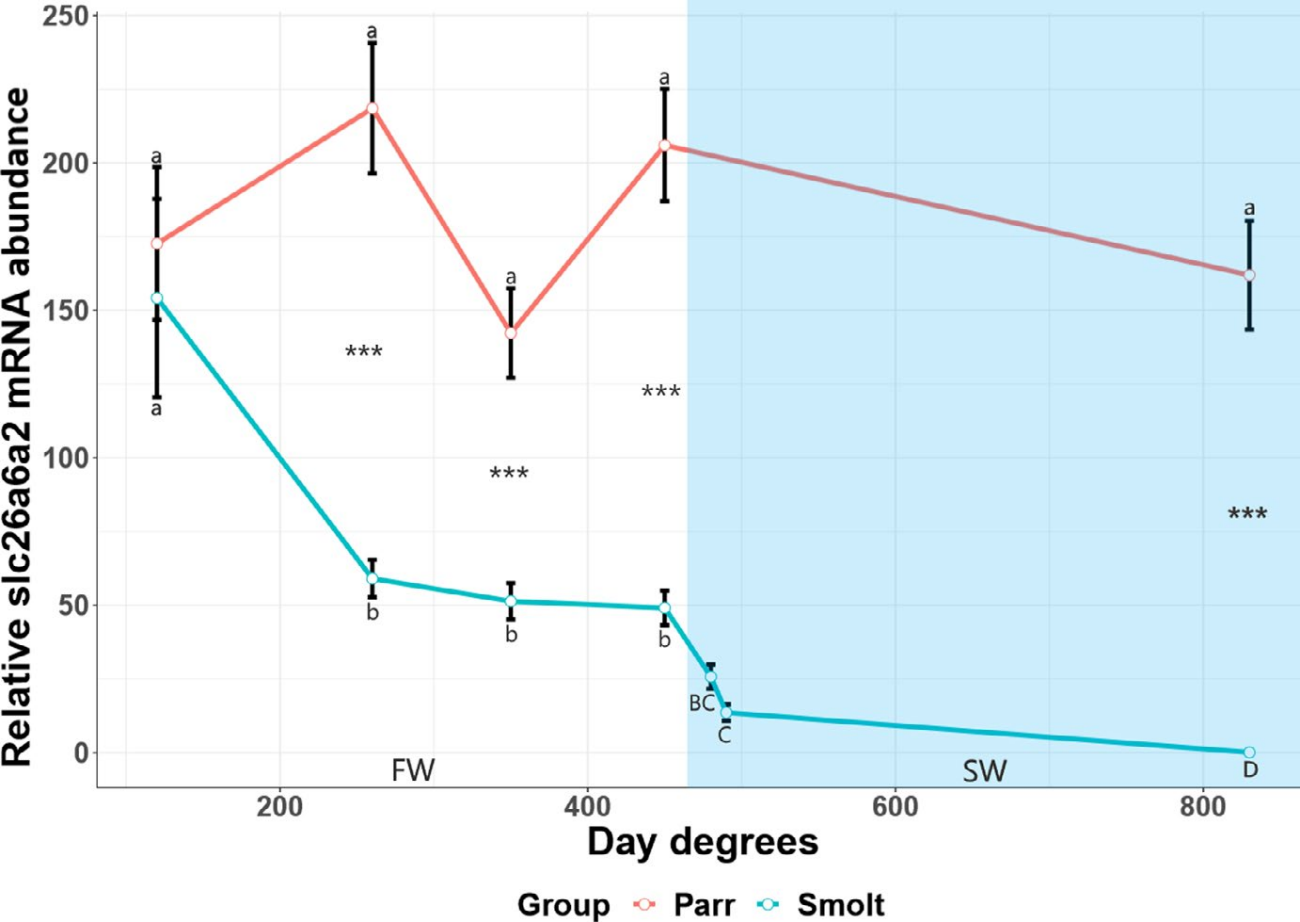
The mRNA abundance of slc26a6a2 in the gills of juvenile Atlantic salmon parr and smolts in freshwater (FW) and smolts after seawater (SW) transfer. Different small letters indicate significant differences between timepoints within the control group (parr) and experimental smolt group in FW (white area of graph), while capital letters indicate differences within each group in SW (blue area of graph). Note that significances following SW transfer are related to last timepoint in FW. Asterisk *p < 0.05, **p < 0.01, and ***p < 0.001 indicate significant differences between groups at each timepoint in both FW and SW. The control group remained in FW during the entire experiment. Each data point is represented as mean ± Standard Error of Mean (SEM) and *n* = 6–10

In the smolt group, *slc26a6a2* expression in gills decreased threefold during smoltification, further decreasing after SW transfer (490 d.d), reaching a 1700‐fold lower expression level after 38 days in SW (830 d.d) compared to first timepoint in FW (Figure 8). In contrast, *slc26a6a2* mRNA abundance did not significantly differ at any timepoint in the parr group (Figure 8) and the smolt group displayed consistently lower *slc26a6a2* mRNA abundance than observed in the parr group (Figure 8).

## 4 DISCUSSION

### 4.1 NKA enzyme activity during smoltification and after SW transfer

The overall decrease in condition factor and increasing smolt index and Nka activity levels in gills and kidney in this study, are consistent with the typical metabolic and physiological changes in smoltifying salmon (Björnsson & Bradley, 2007; Stefansson et al., 2008). Elevated kidney Nka enzyme activity in peak smolts in this study, also reported by McCartney (1976), likely reflect a preparation to meet enhanced requirements for active ion transport before entering the marine environment. High Nka activity is argued to promote and enable reabsorption of roughly 95% NaCl, minimizing salt loss in FW‐acclimated teleosts (Perry et al., 2003; Tang et al., 2010). Despite an increasing kidney Nka activity in FW smolts, the overall high Nka activity in kidney of FW‐acclimated parr emphasizes the importance of a relatively high tubular Nka activity as an direct driver of Na^+^ and indirect driver of Cl^−^ reabsorption in the kidney (Takvam et al., 2021). Changes in kidney Nka enzyme activity can be sensitive and responsive to changes in environmental salinity in other teleosts, with increased Nka activity being linked to a heightened requirement for secretion in SW (Herrera et al., 2009; Madsen et al., 1994). Other reports show no change in Nka activity after SW exposure (Arjona et al., 2007; Sangiao‐Alvarellos et al., 2005), suggesting that at least some species, do not display altered activity in the kidney upon SW exposure. Furthermore, no changes in kidney Nka activity were observed in juvenile salmon parr and smolt gradually exposed to salinities of 10 ppt or 30 ppt over the course of 2 weeks (McCormick et al., 1989), where the differences in either salinity, duration of exposure, and/or developmental stage may explain the different responses reported. The teleost kidney handles Na^+^ and Cl^−^ by secretion in the proximal tubules and reabsorption in the distal tubule and collecting duct (Nishimura et al., 1983; Beyenbach, 1995; Beyenbach et al., 1986; Kato et al., 2011), yet it was not possible to determine if reduction in Nka activity originated from one or more segments of the nephron in this study. To prevent water loss and dehydration in SW, fish rapidly reduce glomerular filtration rates (GFRs) and tubular flow/urine filtration rates (UFRs) in the nephrons during the acute SW phase (Hickman & Trump, 1969; Schmidt‐Nielsen & Renfro, 1975; Brown et al., 1978; Beyenbach, 2004; Takvam et al., 2021). The reduction in filtering nephrons commonly found in previous studies could be correlated with a decrease in tubular Nka activity observed in this study. Ultimately, the transient short‐term reduction in Nka activity in this study indicates a certain requirement for reducing pumping capacity of monovalent ions in nephron tubules and may be a result of rapid reduction of both GFR and UFR in SW. The high Nka enzyme activity after 1 month SW is likely linked to increased requirement to secrete divalent ions in the kidney as previously suggested (Herrera et al., 2009; Madsen et al., 1994). Indeed, the Nka have been highly linked to the transport of sulfate as it produces a negative cytosolic charge that permits sufficient buildup of high cytoplasmic concentrations that can drive apical secretion of ${\text{SO}}_{4}^{2‐}$ by Slc26a6a in SW mefugu. Thus, high enzyme activity levels are required to effectively enable excretion of excess ${\text{SO}}_{4}^{2‐}$ in SW environments (Kato et al., 2009; Watanabe & Takei, 2011a,b).

### 4.2 ${\text{SO}}_{4}^{2‐}$ transporters in Atlantic salmon

Several of the identified ${\text{SO}}_{4}^{2‐}$ transporters in salmon were upregulated in the kidney during smoltification and SW transfer, corresponding with an rapid transient increase in plasma ${\text{SO}}_{4}^{2‐}$ levels before returning to similar levels, indicating kidney‐specific ${\text{SO}}_{4}^{2‐}$ transporters are important for the regulation of ${\text{SO}}_{4}^{2‐}$ plasma levels in SW. Concurrently, a putative gill‐specific solute carrier was highly expressed in FW parr, decreased during smoltification, and further downregulated to undetectable levels after SW transfer, suggesting a role in ${\text{SO}}_{4}^{2‐}$, Cl^−^, or ${\text{HCO}}_{3}^{‐}$ uptake in FW gills. Functional affinity measurement for each of the ions is necessary to ascertain which ion the gill‐specific solute carrier primarily transport.

### 4.3 Characterization of putative ${\text{SO}}_{4}^{2‐}$ transporters in Atlantic salmon

In this study, seven putative ${\text{SO}}_{4}^{2‐}$ transporters were identified and annotated in Atlantic salmon. The solute carrier family 13 member 1 (Slc13a1) was already annotated and additional searches did not reveal any other salmon‐specific paralogues. It is possible that the rediploidization process currently occurring in salmonids (Lien et al., 2016) may have led to a diploid state for the Slc13a1, as approximately 10–20% of the salmon genome still retain residual tetrasomy (Allendorf et al., 2015; Lien et al., 2016). Rediploidization in salmonids suggests retention of about half of the duplicated gene pairs from the salmonid‐specific 4RWGD (Lien et al., 2016), leading to a wider repertoire of gene families than in other teleosts. Our annotations indicate that Atlantic salmon may have retained novel paralogs for both the solute carrier family 26 member 1 (Slc26a1) and member 6 (Slc26a6a). Each of the salmon Slc26a1 paralogs, annotated and termed Slc26a1a and Slc26a1b, grouped closely with their rainbow trout counterparts and the single Northern pike (*Esox lucius*) Slc26a1. The salmon Slc26a1b sequence was originally annotated as Slc26a2 in the salmon genome database. However, synteny analysis using the Genomicus platform (Nguyen et al., 2018), supports re‐annotation of the Slc26a1b. The solute carrier family 26 member 6 orthologue has several teleost‐specific paralogs, annotated as Slc26a6a, Slc26a6b, and Slc26a6c (Kato et al., 2009) and despite bioinformatic sequence assembly may be challenging (Houston & Macqueen, 2019), the phylogenetic (protein sequences) and synteny (gene environment) approaches applied in this study supports our identification of a single salmon Slc26a6b and Slc26a6c sequence and two salmon Slc26a6a paralogs, annotated as Slc26a6a1 and Slc26a6a2.

Genomic duplication events are important mechanisms generating phenotypic diversity (Kellogg, 2003; Kondrashov et al., 2002), and currently three main theories exist concerning the fate of paralog genes; 1) the dosage balance model, 2) sub‐functionalization, and 3) neo‐functionalization (Warren et al., 2014). Non‐functional gene duplicates are often lost during rediploidization. Among the ones that are kept, Lien et al. (2016) argues that Atlantic salmon display more instances of neo‐functionalization than sub‐functionalization. Interestingly, the *slc26a6a1* is only expressed in the kidney and the intestine, while *slc26a6a2* is only found in gills. Furthermore, during smoltification and sea water transfer, *slc26a6a1* is highly regulated in kidneys and *slc26a6a2* in the gill. These regulations are opposite and quite symmetrical, with *slc26a6a1* expression increasing through smoltification being further upregulated after SW transfer, while *slc26a6a2* expression decreasing through smoltification being further downregulated after SW transfer. It can be argued that these salmon‐specific paralogs follow a neo‐functionalization since the tissue distribution and regulation of the *slc26a6a1* are similar to the single *slc26a6a* transporter found in Japanese eel and medaka kidney (Kato et al., 2009; Watanabe & Takei, 2011b), while the *slc26a6a2* is highly regulated in FW and only detected in gills, which is not observed with *slc26a6a* in eel and medaka.

The *slc26a1a* is found in both kidney and intestine while the *slc26a1b* was kidney specific. Compelling evidence suggest that the Slc26a6 family functions as an intestinal HCO_3_^−^ transporter and that intestine is virtually impermeable to ${\text{SO}}_{4}^{2‐}$ (Hickman, 1968; Marshall & Grosell, 2006). However, the intestinal function of the salmon Slc26a1 family need to be determined experimentally before a firm role in HCO_3_^−^ transport can be assigned. The *slc26a1a* and *slc26a1b* are both found in the kidney and regulated in both FW and SW, which support previous studies linking the Slc26a1 family to ${\text{SO}}_{4}^{2‐}$ homeostasis in both FW and SW environments (Kato et al., 2009; Nakada et al., 2005; Watanabe & Takei, 2011b). Regulatory changes observed in this study indicates a sub‐functional regulation (Warren et al., 2014) in Atlantic salmon as they are both detected and regulated in similar tissues to previous studies. Additional functional studies of the Slc26a6a1 (kidney and intestine), Slc26a6a2 (gills), Slc26a1a (kidney and intestine), and Slc26a1b (kidney) paralogs are required to further characterize the physiological properties of these transporters.

In this study, several predicted splice variants were identified and their expression patterns were examined. Alternative splicing or differential splicing, is a critical regulatory process that permits a single gene to code for multiple proteins in biological systems (Kim et al., 2008; Wang et al., 2015). Alternative splicing variants found in fugu (*Takifugu rubripes*), medaka (*Oryzias latipes*), and zebra fish (*Danio rerio*) are proposed to be important for the functional and evolutionary mechanisms of genomes in teleost fish (Lu et al., 2010). It has been proposed that alternative splice variants may be activated in the process of adapting to altered salinities or other challenging events (Kijewska et al., 2018). In this study, the kidney‐specific *slc26a1bX3* splice variant and the kidney and intestinal *slc26a1bX1* splice variant displayed similar expression patterns in FW‐ and SW‐acclimated salmon, while the *slc26a1bX2* splice variant was not expressed in tissues studied. Of the *slc26a6a2X1* and *slc26a6a2X2* splice variants, only the *X1* was expressed (gills) while *X2* was not expressed in tissues studied. We cannot rule out that splice variants *slc26a6a2X2* and *slc26a1bX2* may be specific for other tissues not investigated in this study. The splicing mechanisms of mRNAs are, however, complex and despite identification of putative splice variants for *slc26a6a2* (*slc26a6a2X1* and *slc26a6a2X2*) and *slc26a1b* (*slc26a1bX1*, *slc26a1bX2*, and *slc26a1bX3*) suggests high regulatory plasticity in Atlantic salmon, no further conjecture is formulated as it goes beyond the data and scope of this study.

### 4.4 Regulation of ${\text{SO}}_{4}^{2‐}$ transporters in Atlantic salmon

#### 4.4.1 Kidney is the main regulator of ${\text{SO}}_{4}^{2‐}$ in fish

In mammals, the SLC26A6 transporter has been localized to apical membranes in proximal tubules of the kidney and proposed to exchange numerous anions: oxalate/${\text{SO}}_{4}^{2‐}$, Cl^−^/formate, Cl^−^/oxalate, oxalate/formate, oxalate/oxalate, Cl^−^/HCO3^−^, and Cl^−^/OH^−^ (Markovich, 2001; Markovich & Aronson, 2007), while the SLC26A1 (SAT‐1) is a ${\text{SO}}_{4}^{2‐}$/anion exchanger, mediating ${\text{SO}}_{4}^{2‐}$ efflux across the basolateral membrane in exchange of HCO_3_^−^ (Karniski et al., 1998). In the teleost kidney, the prevailing hypothesis has largely been its apparent ability of ${\text{SO}}_{4}^{2‐}$ transport to be directed via a Cl^−^ gradient (Renfro et al., 1999; Renfro & Pritchard, 1983), facilitated through the apical Slc26a6 and the basolateral Slc26a1 transporters (Kato et al., 2009; Watanabe & Takei, 2011a,b).

Electrophysiological studies of teleost sequences in Xenopus oocytes revealed a 50‐ to 200‐fold higher electrogenic transport by the Slc26a6a than the Slc26a6b paralogs, with the Slc26a6a displaying the highest ${\text{SO}}_{4}^{2‐}$ transport activity among the Slc26a6 family (Kato et al., 2009; Watanabe & Takei, 2011b). These studies largely suggest that a negative cytosolic charge powered by the Nka enzyme yields low cytoplasmic Cl^−^ concentrations via chloride channels, aided by the basolateral ${\text{SO}}_{4}^{2‐}$/HCO_3_^−^ exchanger Slc26a1. This permits sufficient buildup of high cytoplasmic concentrations of ${\text{SO}}_{4}^{2‐}$ driving apical secretion of ${\text{SO}}_{4}^{2‐}$ by Slc26a6a in SW mefugu and eel (Kato et al., 2009; Watanabe & Takei, 2011a,b). Upregulation during smoltification and the rapid increase after 2 days of SW exposure, further increasing after more than 1 month in SW in this study suggest an important role of the *slc26a6a1* in secreting excess ${\text{SO}}_{4}^{2‐}$ in SW‐acclimated salmon. The *slc26a6a1* mRNA abundance was barely detectable in FW parr, while increased *slc26a6a1* levels in FW smolts suggest that this transporter is not merely activated by salinity, as previously suggested in eel and mefugu (Kato et al., 2009; Watanabe & Takei, 2011a), but are rather regulated as the smolt prepare for entering SW. Similar patterns are found for the *slc26a1a* paralog, as it is substantially upregulated in FW smolt compared to the parr group, hence not fully elevated until 1 month in SW. The *slc26a6b* and *slc26a6c* paralogs in teleosts are upregulated in both FW and SW and are linked to apical transport of ${\text{SO}}_{4}^{2‐}$ in the renal proximal tubule I and II (Kato et al., 2009; Watanabe & Takei, 2011b). In this study, a similar expression pattern of *slc26a6b* and *slc26a6c* in both parr and smolt, as well as FW and SW environments, suggests that both paralogs are indeed active in both FW and SW. Slc26a6b is suggested to be a Cl^−^/${\text{SO}}_{4}^{2‐}$ anion exchanger, similar to the Slc26a6a, while electrophysiological studies suggest Slc26a6c is not an anion exchanger (Kato et al., 2009). Based on the expression patterns of salmon *slc26a6b* and *slc26a6c* in FW and SW, it can be argued that these transporters may have dual roles, reabsorbing in FW and secreting in SW. Still, further verification at the protein level is required before firm conclusions can be made with respect to localization and function of the Slc26a6b and Slc26a6c in Atlantic salmon.

The upregulation of *slc26a6a1* in smolts after short‐ and long‐term SW exposure infers an important role for salmon in SW, similar to what has been demonstrated for other species upon SW transfer (Kato et al., 2009; Watanabe & Takei, 2011). There is a common consensus that most ${\text{SO}}_{4}^{2‐}$ is actively secreted from the renal proximal tubules of marine teleost and euryhaline species in SW to maintain plasma ${\text{SO}}_{4}^{2‐}$ levels within 0.2–2 mM, and that the urine is rich in ${\text{SO}}_{4}^{2‐}$ ions (roughly 45–50 mM) (Hickman & Trump, 1969; Renfro, 1999; Beyenbach, 2004; Marshall & Grosell, 2006; Watanabe & Takei, 2011b, 2012). Plasma levels of ${\text{SO}}_{4}^{2‐}$ range between 0.1–0.3 mM in FW and 0.8–1.2 mM in SW‐acclimated salmonids, respectively (Katoh et al., 2006; Watanabe & Takei, 2012). In FW‐acclimated rainbow trout, in vitro injections of ${\text{SO}}_{4}^{2‐}$ resulted in a substantial increase in plasma ${\text{SO}}_{4}^{2‐}$ levels from 0.45 mM (base level) to 2.25 mM, followed by a subsequent rapid return to 0.6 mM, reflecting efficient regulation of plasma ${\text{SO}}_{4}^{2‐}$ concentrations, mainly by the kidney (Katoh et al., 2006). In this study, plasma ${\text{SO}}_{4}^{2‐}$ levels ranged between 0.6 and 0.8 mM in FW‐acclimated parr and smolt, followed by a spike in plasma ${\text{SO}}_{4}^{2‐}$ levels to 1–1.8 mM after 2 days in SW, which is somewhat lower than in rainbow trout. Such differences could be species specific, but also due to 2Na^+^ ${\text{SO}}_{4}^{2‐}$ being directly injected in rainbow trout, probably leading to an instant rise in plasma ${\text{SO}}_{4}^{2‐}$ (Katoh et al., 2006). Despite an unavoidable influx of ${\text{SO}}_{4}^{2‐}$ probably occurs through the gills in SW teleosts (Watanabe & Takei, 2012), smolts in SW experiences a minor rise in plasma ${\text{SO}}_{4}^{2‐}$ levels, consistent with their preparatory upregulation of sulfate transporters. About 97% of the ${\text{SO}}_{4}^{2‐}$ is excreted via the kidney in SW environments (Watanabe & Takei, 2012) which is consistent with mRNA expression of the putative secretory ${\text{SO}}_{4}^{2‐}$ transporters in this study (*slc26a6a1 and slc26a1a)* and reports on the Slc26a6a and Slc26a1 in Japanese eel, mefugu, and rainbow trout (Kato et al., 2009; Katoh et al., 2006; Watanabe & Takei, 2011a, 2011b). The above clearly suggests that excess ${\text{SO}}_{4}^{2‐}$ is secreted through the nephron tubule in fish where both the Slc26a6a and Slc26a1 family play a significant role, probably in conjunction with the driving force of the Nka enzyme (Figure 1b), as reflected by both plasma (Figure 2) and mRNA levels (Figure 6). The current spike in ${\text{SO}}_{4}^{2‐}$ plasma levels after 2 days correlate well with an increase in *slc26a6a1*, the most plausible candidate as an apical secretory ${\text{SO}}_{4}^{2‐}$ transporter (Slc26a6a; Kato et al., 2009; Watanabe & Takei, 2011a,b). Furthermore, the increase in the *slc26a6a1* (secretory, presumably apical) and *slc26a1a* (the most plausible basolateral ${\text{SO}}_{4}^{2‐}$ transporter) (Kato et al., 2009; Watanabe & Takei, 2011b) after 1 month in SW suggest a combined effort for these transporters to effectively remove ${\text{SO}}_{4}^{2‐}$ in SW. In this study, a short‐term concurrent decrease in kidney Nka enzyme activity following short‐term SW exposure is consistent with the apparent need for conserving water, which likely is a response to reduce filtration rates and tubular activity in the kidney, while the increased activity observed in long‐term SW acclimation are probably required to secrete excess divalent ions. These assumptions are strengthened by the apparent role of the NKA pump, hypothesized as the main driving force for ${\text{SO}}_{4}^{2‐}$ transport in teleosts (Kato et al., 2009; Watanabe & Takei, 2011b).

Direct transfer to full strength SW results in a significant transient spike in ${\text{SO}}_{4}^{2‐}$ plasma levels, a concurrent transient short‐term decrease in Nka activity and rapid upregulation of the secretory *slc26a6a1* transporter while the *slc26a1a* remains relatively stable. This indicates a short‐term disturbance in the transport activity (reflected in Nka activity levels) and a need to remove excess ${\text{SO}}_{4}^{2‐}$ from the plasma (increase in *slc26a6a1*) in salmon smolts immediately following SW transfer. Sustained expression levels of *slc26a6a1* and *slc26a1a* and increase in kidney Nka enzyme activity concurrent with plasma ${\text{SO}}_{4}^{2‐}$ levels returning to normal after 1 month in SW suggest that salmon smolts require more than 2 days to fully acclimate and effectively remove excess ${\text{SO}}_{4}^{2‐}$ ions from plasma. In addition, almost 325 times higher ${\text{SO}}_{4}^{2‐}$ concentration in SW (33 mM) than FW (0.1 mM) results in production of urine rich in ${\text{SO}}_{4}^{2‐}$ ions (45–50 mM) (Hickman & Trump, 1969; Watanabe & Takei, 2012), emphasizing the requirement of an efficient and sophisticated transport pathway to remove ${\text{SO}}_{4}^{2‐}$ in the kidney. We hypothesize that the *slc26a6a1* and *slc26a1a* are the most likely candidate for ${\text{SO}}_{4}^{2‐}$ excretion in salmon which is further substantiated by detailed electrophysiological and molecular investigations in SW mefugu, eel, and rainbow trout (Kato et al., 2009; Katoh et al., 2006; Watanabe & Takei, 2011a,b).

**FIGURE 9:**
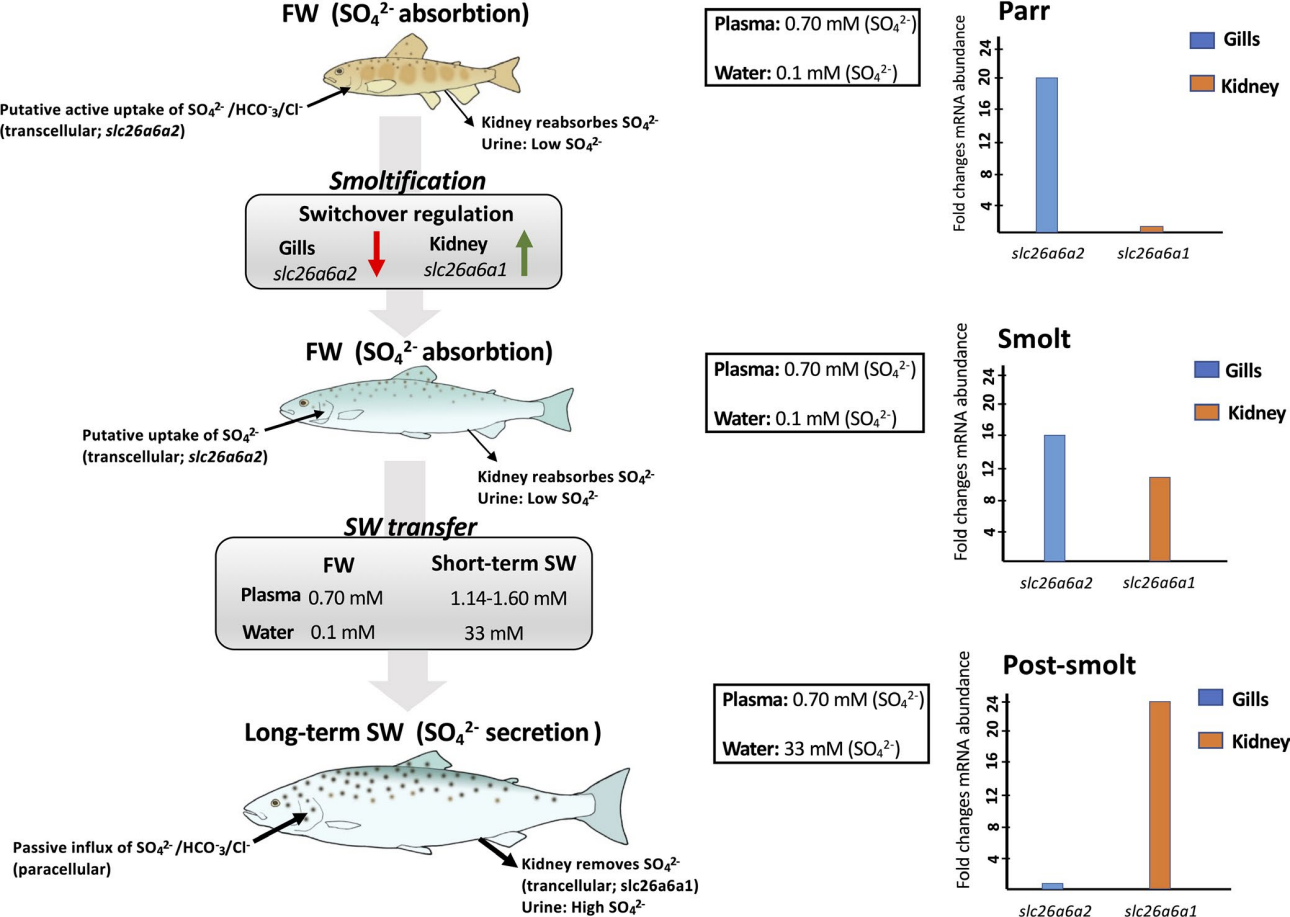
Sulfate homeostasis during smoltification and SW exposure is associated with differential regulation of paralog transporters in gills and kidney. In FW parr and smolts the gill may absorb sulfate (${\text{SO}}_{4}^{2‐}$) aided by the kidney, which reabsorbs ${\text{SO}}_{4}^{2‐}$ resulting in low urine ${\text{SO}}_{4}^{2‐}$ concentrations in order to maintain a sevenfold higher plasma levels (0.70 mM) compared to FW (0.1 mM). During smoltification the secretory slc26a6a1 ${\text{SO}}_{4}^{2‐}$ transporter increases 11‐fold in kidney that indicate the important switch in regulation to prepare for ${\text{SO}}_{4}^{2‐}$ excretion in SW. In short‐term SW there is a spike in ${\text{SO}}_{4}^{2‐}$ plasma levels in parallel with a further increase of the secretory kidney slc26a6a1. In long‐term SW the plasma levels return to normal ${\text{SO}}_{4}^{2‐}$, similar to that of FW fish, in which the secretory kidney slc26a6a1 transporter increases further about 20‐fold higher that FW parr levels. The passive influx of ${\text{SO}}_{4}^{2‐}$ via the gills from the SW environment (33 mM) is effectively removed by the kidney via the slc26a6a1 (possibly also slc26a1a, not shown) to excrete high levels of ${\text{SO}}_{4}^{2‐}$ through the urine to maintain normal plasma levels (0.69 mM). In the gills, the slc26a6a2 decrease threefold during smoltification and are further decreased to undetectable levels about 2000‐fold lower that FW parr levels. The slc26a6a2 may be a putative absorptive transporter for either ${\text{SO}}_{4}^{2‐}$, HCO_3_^−^, or Cl^−^ where affinity measurements of the three ions are necessary to find the specific function of the paralog. The proposed model is also based and substantiated by previous investigation on ${\text{SO}}_{4}^{2‐}$ regulation in fish (Kato et al., 2009; Watanabe & Takei, 2011a, 2011b, 2012)

Despite our suggested model of ${\text{SO}}_{4}^{2‐}$ transport in the Atlantic salmon kidney is premature (Figure 9), future studies on all gene candidates, particular the salmon‐specific paralogs are vital to fully elucidate the regulation and physiological properties of these transporters in Atlantic salmon. Hence, characterization of a complete transport model requires comprehensive studies of cellular localization and co‐transport with other ion transporters to fully elucidate the transport mechanisms. We suggest that the Slc26a6a1 and Slc26a1a transporters are important for tight regulation of plasma ${\text{SO}}_{4}^{2‐}$ levels in response to the substantial difference in ${\text{SO}}_{4}^{2‐}$ concentrations in FW (0.01 mM) to SW (30 mM). However, one cannot exclude a possible role for the Slc26a6b, Slc26a6c, Slc26a1bX1, and Slc26a1bX3 in ${\text{SO}}_{4}^{2‐}$ homeostasis as they likely perform tasks in both FW and SW environments.

### 4.5 Possible involvement of gill‐specific Slc26a6a2 paralog in ion uptake (${\text{SO}}_{4}^{2‐}$/HCO_3_^−^/Cl^−^) in FW

The putative *slc26a6a2* paralog was by far the most abundantly expressed transporter in FW‐acclimated salmon, followed by threefold decrease during smoltification, and an 1800‐fold downregulation in the smolt following long‐term SW exposure, suggesting a role in ion uptake across the gills in FW‐acclimated salmon.

It has been suggested that the teleost gill may be a site for ${\text{SO}}_{4}^{2‐}$ absorption in FW‐acclimated fish (Watanabe & Takei, 2012). Furthermore, a low influx of ${\text{SO}}_{4}^{2‐}$ from the medium to the body (0.09 µmol/kg/h) has been observed in FW teleosts, indicating low permeability and minimal paracellular transport. Additionally, FW fish usually do not drink while in freshwater and possible absorption through the intestine are minuscule (see discussion intestine). Plasma ${\text{SO}}_{4}^{2‐}$ concentrations in this study are about sevenfold higher than that of the surrounding FW, suggesting active ${\text{SO}}_{4}^{2‐}$ uptake, potentially across the gills. To date, no potential candidates have been suggested but, here we hypothesis that transcellular transport against an electrochemical gradient is possible *via* Slc26a6a2, probably driven by the NKA pump. The SLC26A6A family can have several potential transporter roles in mammals, such as oxalate/${\text{SO}}_{4}^{2‐}$, Cl^−^/formate, Cl^−^/oxalate, oxalate/formate, oxalate/oxalate, Cl^−^/HCO3^−^, and Cl^−^/OH^−^ (Markovich, 2001). In teleost fishes, the Slc26a6a transporter has been accredited the following roles: in the kidney increasing evidence points to a role in ${\text{SO}}_{4}^{2‐}$ transport (see discussion kidney) and in the intestine most evidence points to a role in HCO_3_^−^ transport (see discussion intestine). However, determining the role of the Slc26a6a2 in the gills is challenging as the SLC26 family may be involved in transport of several ions (Cl^−^ and HCO_3_^−^) in FW‐acclimated teleosts (Deigweiher et al., 2008; Evans et al., 2005; Leguen et al., 2015; Perry & Gilmour, 2006). Additionally, the striking sequence similarity between Slc26a6a1 and Slc26a6a2 paralogs and differential regulation in different tissues and environments adds to the complexity. Nevertheless, the current regulation of *slc26a6a2* most certainly suggest an importance for ion uptake (${\text{SO}}_{4}^{2‐}$/HCO_3_^−^/Cl^−^) in FW‐acclimated fishes (Figure 9). Therefore, it will be important to both determine the cellular location and the specific affinity of paralog Slc26a6a2 in relation to the different ions (${\text{SO}}_{4}^{2‐}$/HCO_3_^−^/Cl^−^) as the SLC26A6 family appears to have a broad ion specificity in both fish and mammals alike.

### 4.6 The intestine has a lesser role in ${\text{SO}}_{4}^{2‐}$ transport

The intestine contributes less to the overall ${\text{SO}}_{4}^{2‐}$ budget in SW teleosts, with roughly 15% uptake through the intestinal tract (Watanabe & Takei, 2012). Furthermore, up to 85% of ${\text{SO}}_{4}^{2‐}$ uptake originates from gills/skin and are almost exclusively secreted by the kidney (97%) in SW. The intestinal fluid is rich in ${\text{SO}}_{4}^{2‐}$ and the intestine of marine teleosts is believed to be almost impermeable to ${\text{SO}}_{4}^{2‐}$ (Hickman, 1968; Marshall & Grosell, 2006). This is somewhat contradictory since both Cl^−^ and HCO_3_^−^ appear to influence ${\text{SO}}_{4}^{2‐}$ transport in the intestine of marine teleosts (Grosell, 2010; Pelis & Renfro, 2003). Thus, the transport activity of ${\text{SO}}_{4}^{2‐}$ is generally low in the intestine and reflects the high concentrations of ${\text{SO}}_{4}^{2‐}$ in intestinal fluids of marine fish (Grosell, 2010; Hickman, 1968; Marshall & Grosell, 2006). Increasing evidence points to the Slc26a6 family as an intestinal Cl^−^/HCO_3_^−^ exchanger in marine teleosts and SW‐acclimated euryhaline teleost (Kurita et al., 2008; Sundell & Sundh, 2012; Wilson et al., 2002). This study demonstrated that *slc13a1*, *slc26a6a1*, *slc26a6b*, *slc26a1a*, and *slc26a1bX1*, are all expressed in salmon intestine. Among these, members of the Slc26a6 family, represented by the Slc26a6a and Slc26a6b paralogues, are the only ones verified as potential HCO_3_^−^ transporters in teleosts to date (Kurita et al., 2008). Regulation of *slc26a6a* and *slc26a6b* in FW‐ and SW‐acclimated salmon is similar to expression patterns in euryhaline mefugu (Kurita et al., 2008). It remains to determine the function of Slc26a1a, Slc26a1bX1, and Slc13a1 in salmon intestine, yet given that the intestine may be impermeable to ${\text{SO}}_{4}^{2‐}$ and the Slc26 family is linked to intestinal HCO_3_^−^ regulation, the intestinal function is probably HCO_3_^−^ transport rather than ${\text{SO}}_{4}^{2‐}$ transport.

### 4.7 Summary and future perspective

Searches in the salmon genome and phylogenetic analysis revealed annotated and non‐annotated sequences of solute carrier family 13 (Slc13) and 26 (Slc26), including: Slc13a1 (intestine), Slc26a6a (gills, intestine, and kidney), Slc26a6b (intestine and kidney), Slc26a6c (kidney), and Slc26a1 (intestine and kidney). Salmon‐specific paralogues of Slc26a6a (Slc26a6a1 and Slc26a6a2) and Slc26a1 (Slc26a1a and Slc26a1b) are retained after the salmonid‐specific fourth vertebrate whole genome duplication, and their tissue‐specific expression and regulation suggest neo‐functionalization (Slc26a6a family) and sub‐functionalization (Slc26a1 family), respectively. The preparatory increase in kidney‐specific *slc26a6a1* and *slc26a1a* mRNA levels, in addition to the gill‐specific decrease of *slc26a6a2* expression during smoltification and SW transfer may suggest an important role of these sulfate transporters in the regulatory shift from absorption to secretion moving from FW to SW in the kidney (Figure 9). However, affinity measurements of different ions (${\text{SO}}_{4}^{2‐}$/HCO_3_^2−^/Cl^−^) are required before firm conclusions regarding the role of Slc26a6a2 in the gills. The expression of the *slc26a6b*, *slc26a6c*, and *slc26a1b* remained stable, with no significant differences between parr and smolts, suggesting dual roles, thus being active in both FW‐ and SW‐acclimated fish. The expression of salmon Slc26a1 and Slc26a6 families in the kidney, gills, and intestine, probably reflect a broad ion specificity. However, this study supports the vital role of the kidney in ${\text{SO}}_{4}^{2‐}$ excretion through the highly upregulated *slc26a6a1*, the most likely secretory transport candidate in fish, which together with the *slc26a1a* transporter likely removes excess ${\text{SO}}_{4}^{2‐}$, mitigating passive influx through the gills and ultimately enable regulation of normal plasma ${\text{SO}}_{4}^{2‐}$ levels in SW (Figure 9). Our findings emphasize a highly effective strategy in which the Slc26a1 and Slc26a6 families likely perform different tasks depending on the tissue in which they are expressed. Thus, the neo‐functionalization of the *slc26a6a1* (kidney) and *slc26a6a2* (gills) may have provided the salmon a notable plasticity in regulating ions effectively when migrating between FW and SW habitats. Immunolocalization and precise affinity measurements of the described ${\text{SO}}_{4}^{2‐}$ transporters are required to further our understanding on how ${\text{SO}}_{4}^{2‐}$ homeostasis is regulated in teleost fish.

## CONFLICT OF INTEREST

The authors declare no conflict of interest.

## AUTHOR CONTRIBUTIONS

Tom O. Nilsen (TON) and Marius Takvam (TK) conceived and performed the study. TK, Elsa Denker (ED), and Naoul gharbi (NG) analyzed the samples. Harald Kryvi (HK) made the drawings. All authors contributed to data analysis. TK and TON drafted the manuscript, and all authors reviewed and edited the manuscript.

## Supporting information



Fig S1‐S6

Table S1‐S3
