# Supplementary material for: Sulfate homeostasis in Atlantic salmon is associated with differential regulation of salmonid‐specific paralogs in gill and kidney
Source: Physiol Rep. 2021 Oct 7;9(19):e15059. doi: 10.14814/phy2.15059 (PMC8495805; doi:10.14814/phy2.15059)
Supplement: Supplementary file 1 — Fig S1‐S6 [file PHY2-9-e15059-s002.docx]

**Smolt characteristics and growth measurements**

**
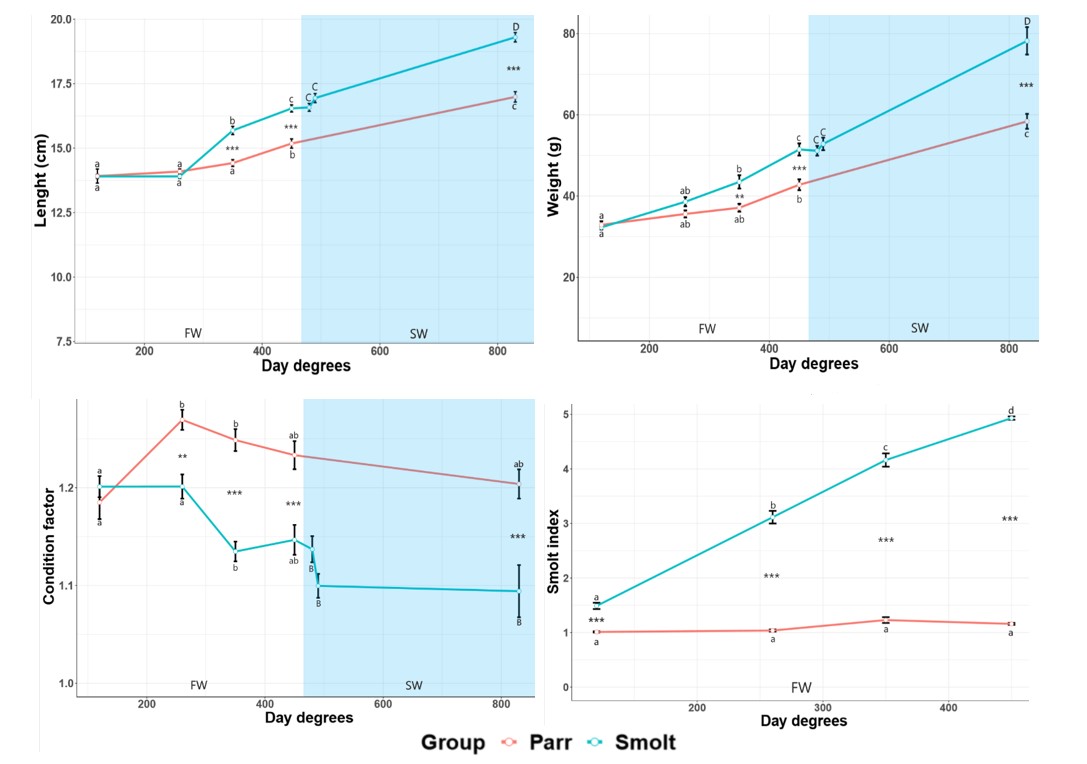
**

**Supplementary Figure 1: Fork length (centimeters, cm), weight (grams, g), smolt index and condition factor of juvenile Atlantic salmon parr and smolts in freshwater (FW) and smolts after seawater (SW) transfer**. *Different small letters indicate significant differences between timepoints within the control group (parr) and experimental smolt group in FW (white area of graph), while capital letters indicate differences within each group in SW (blue area of graph). Note that significances following SW transfer is related to last time-point in FW. Asterisk * p<0,05; **p<0,01 and ***p<0,001 indicate significant differences between groups at each time-point in both FW and SW. The control group remained in FW during the entire experiment. Each data point is represented as mean ± Standard Error of Mean (SEM) and n=10-12.*

**Refining Nka activity method for the kidney**
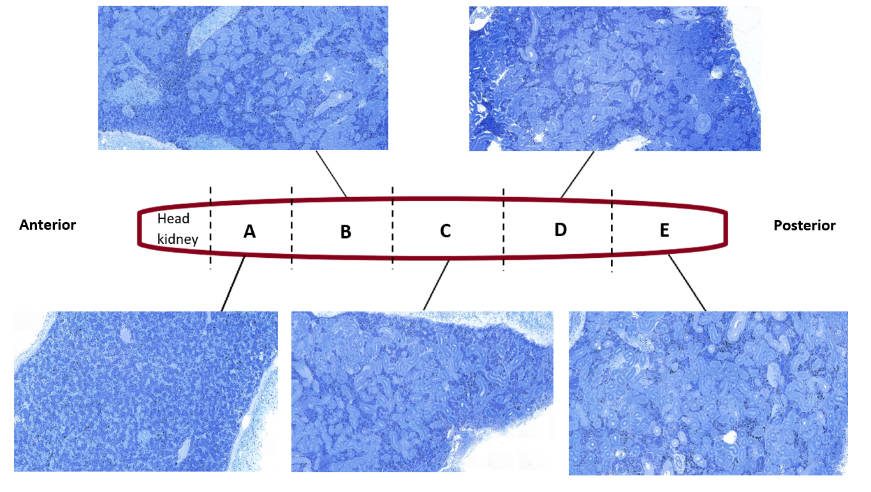


**Supplementary Figure 2. Overview showing the different sections of the kidney.** *Kidney tissue sections stained with toluidine blue allowed visualisation of nephron density and hematopoietic tissue in the kidney. The head kidney was excluded for sampling and five equal sections was obtained from anterior to posterior (A to E) in the SW acclimated Atlantic salmon (n=3) in which an increasing nephron density was observed. The D and E area was selected and used during sampling of kidney tissue.*


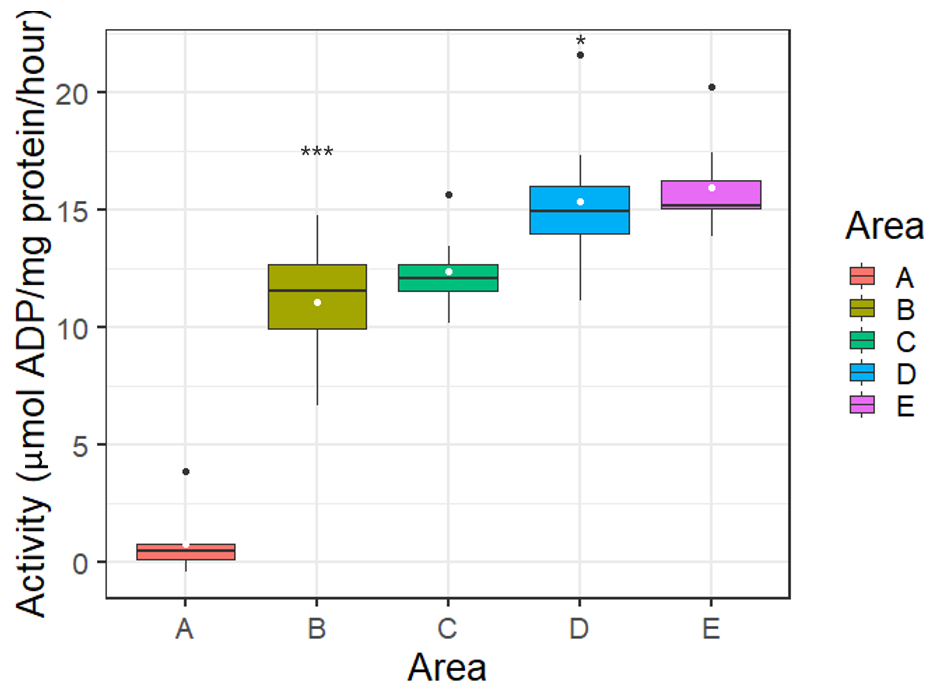


**Supplementary Figure 3: Distribution of Nka activity in five areas in the kidney of SW Atlantic salmon.** Kidney Nka activity levels (µmoles ADP/mg protein/hour) of SW acclimated Atlantic salmon (n=3). Note that significant differences are related to last preceding datapoint from A to E. Asterix * p<0.05; **p<0.01 and ***p<0.001 indicates significant differences between areas in the kidney. Each datapoint (Area) is represented as mean ± standard error of mean (SEM) and n=24. The D and E area was selected and used for sampling of kidney tissue.


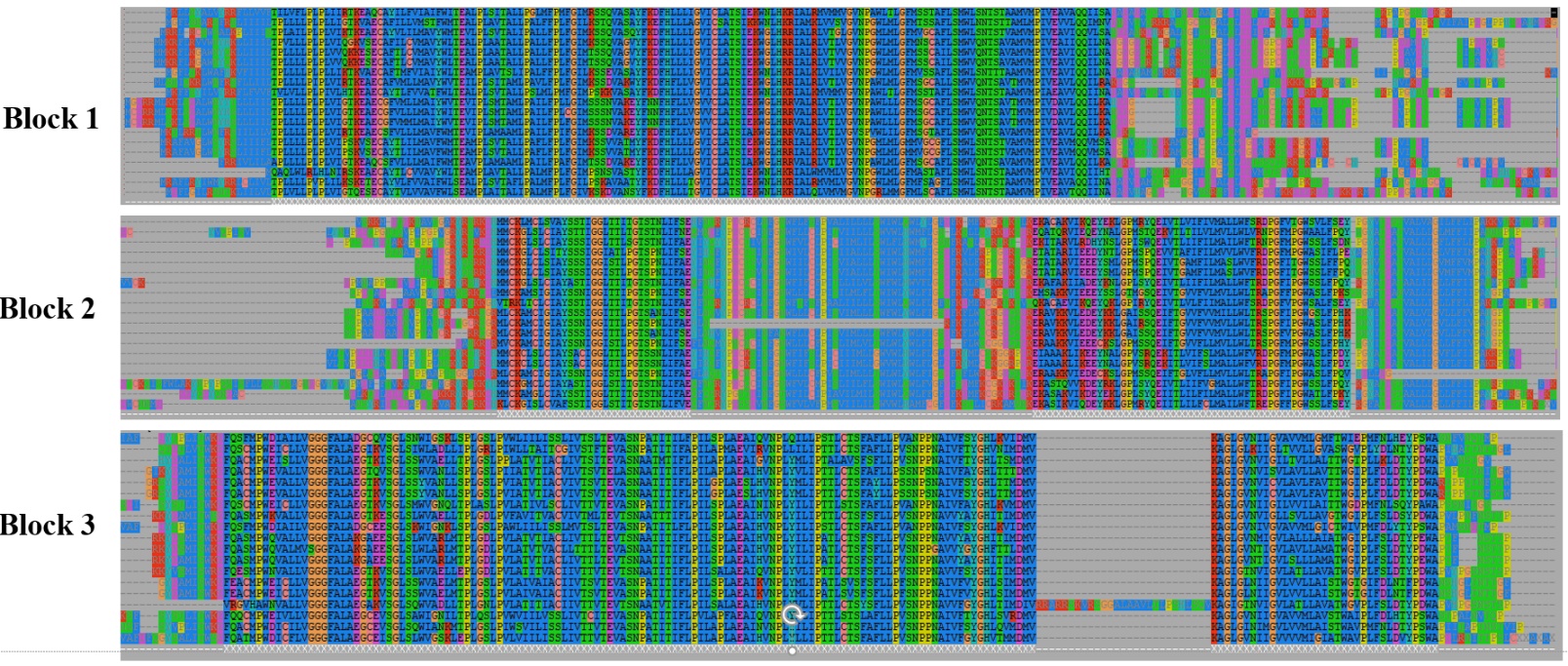
**Protein sequences and alignments for sulfate transporters**

**Supplementary Figure 4: Putative protein sequences, Solute Carrier Family 13 Member 1, Slc13a1.** *The sequences were retrieved from the NCBI database and aligned using Seaview. The GBLOCK function has removed non-conserved positions (faded in color), leaving conserved domains (not faded) to build a phylogenetic tree. The complete sequence is read from left to right for each block (1-3) and the following 19 species are located from top to bottom within each block: Mus musculus, Anguilla japonica, Danio rerio, Salmo salar (1), Salmo salar (2), Salmo salar (3), Lepistoutus oculuctus, Orizias latipes , Homo sapiens, Oreochromis niloticus (1), Oreochromis niloticus (2), Oreochromis niloticus (3), Tetraodon nigroviridis, Latimera chalumnae, Xenopus tropicalis and Callorhinchus milli. Identical sequences were removed leaving 16 out of 19 potential protein sequences for the construction of the phylogentic tree (see figure 5, main text).*


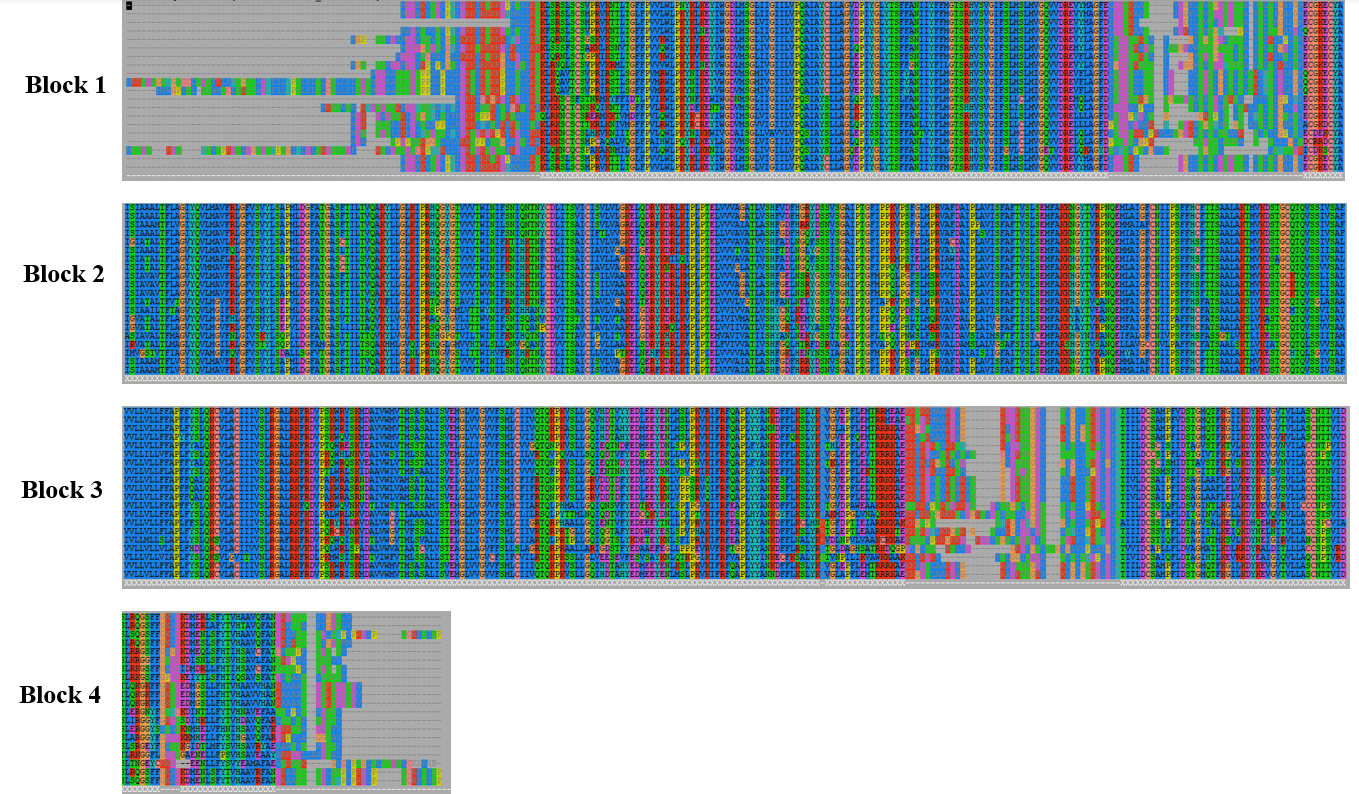


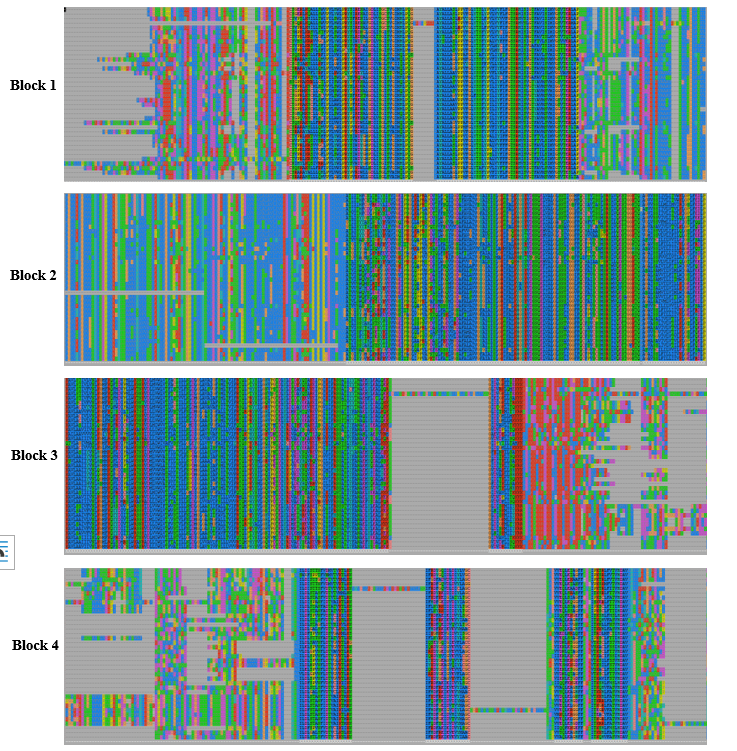
**Supplementary Figure 5: Putative protein sequences, Solute Carrier Family 26 member 1, Slc26a1.** *The sequences were retrieved from the NCBI database and aligned using Seaview. The GBLOCK function has removed non-conserved positions (faded in color), leaving conserved domains (not faded) to build a phylogenetic tree. The complete sequence is read from left to right for each block (1-4) and the following 20 species are located from top to bottom within each block: Salmo salar (1), Oncorhynchus mykiss (1), Anguilla japonica, Esox lucius, Carassius auratus, Lepistoutus oculuctus, Danio rerio, Astyanax mexicanus, Oreochromis niloticus (1), Oreochromis niloticus (2), Oreochromis niloticus (3), Latimera chalumnae, Xenopus tropicalis, Anolis carolinensis, Pelodiscus sinensis, Callorhinchus milli, Mus musculus, Homo sapiens, Salmo salar (2) and Oncorhynchus mykiss (2). Identical sequences were removed leaving 18 out of 20 potential protein sequences for construction of the phylogentic tree (see figure 6, main text).*

**Supplementary Figure 6: Putative protein sequences, Solute Carrier Family 26 member 6, Slc26a6.** *The sequences were retrieved from the NCBI database and aligned using Seaview. The GBLOCK function has removed non-conserved positions (faded in color), leaving conserved domains (not faded) to build a phylogenetic tree. The complete sequence is read from left to right for each block (1-4) and the following 38 species are located from top to bottom within each block: Takifugu obscurus (1), Takifugu rubripes, Orizias latipes, Tetraodon nigrovirid, Oreochromis niloticus, Salmo salar (1), Esox Lucius, Astyanax mexicanus*,  *Oncorhynchus mykiss (1), Oncorhynchus mykiss (2),* *Callorhinchus milli , Mus musculus, Xenopus tropicalis*, *Homo sapiens*, *Latimera chalumnae, Salmo salar (1), Takifugu obscurus (2), Salmo salar (2), Oreochromis niloticus, Esox lucius (2), Carassius auratus, Danio rerio, Oncorhynchus mykiss (3), Haplochromis burtoni, Astyanax mexicanus, Takifugu obscurus (3), Oreochromis niloticus (2), Salmo salar (3), Carassius auratus (2), Danio rerio (2), Oncorhynchus mykiss (4), Haplochromis burtoni (2). Identical sequences were removed leaving 32 out of 38 potential protein sequences for construction of the phylogentic tree (see figure 7, main text).*
