# Supplementary material for: Sulfate homeostasis in Atlantic salmon is associated with differential regulation of salmonid‐specific paralogs in gill and kidney
Source: Physiol Rep. 2021 Oct 7;9(19):e15059. doi: 10.14814/phy2.15059 (PMC8495805; doi:10.14814/phy2.15059)
Supplement: Supplementary file 2 — Table S1‐S3 [file PHY2-9-e15059-s001.docx]

**Supplementary Table 1.** *Overview of the smolt score criteria*

**
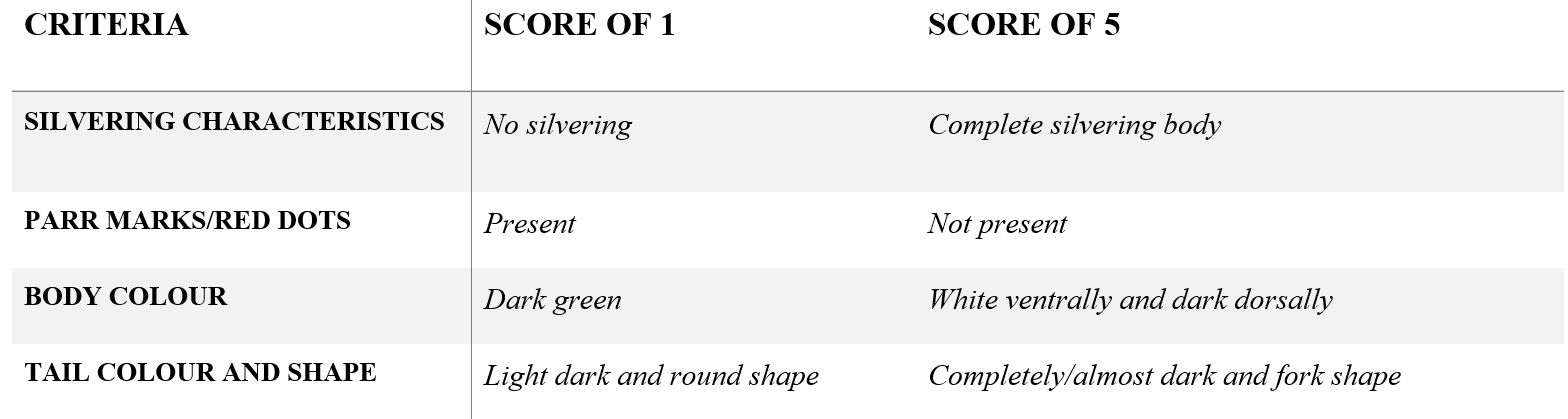
**

**Supplementary Table 2:** *Overview all protein sequences for transport family Slc13a1, Slc26a6a, Slc26a6b, Slc26a6c and Slc26a1. The table includes species (reference and target), protein reference, tissue localization, subcellular localization, water condition, protein name, ref and locus (only for target species, Atlantic salmon) and verification by synteny*.


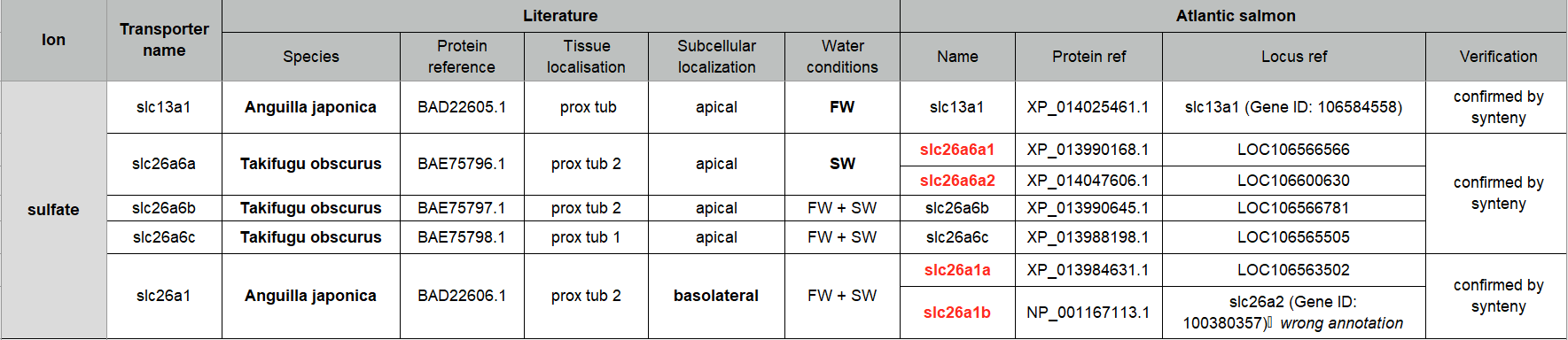


**Supplementary Table 3.** *Overview of all forward (F) and reverse (R) primers designed for putative solute carrier family 26 member 1 (slc26a1a, slc26a1b) and member 6 (slc26a6a1, slc26a6a2, slc26a6b, slc26a6c) and family 13 member 1 (slc13a1) in the Atlantic salmon. The table includes gene name, primers sets, sequence (5` to 3` direction), Amplicon length (nucleotides), position (exon (ex) junctions (jct)), primer length (nucleotides), GC content (%), melting temperature (Tm; basic, salt adjusted, nearest neighbor), primer dimer formation (Hairpin, Complementarity, Self-annealing) and Ta (Annealing temperature).*
